# *VaAPL1* Promotes Starch Synthesis to Constantly Contribute to Soluble Sugar Accumulation, Improving Low Temperature Tolerance in Arabidopsis and Tomato

**DOI:** 10.3389/fpls.2022.920424

**Published:** 2022-06-22

**Authors:** Guoping Liang, Yanmei Li, Ping Wang, Shuzhen Jiao, Han Wang, Juan Mao, Baihong Chen

**Affiliations:** College of Horticulture, Gansu Agricultural University, Lanzhou, China

**Keywords:** grape, *VaAPL1*, low temperature, *Arabidopsis thaliana*, tomato, transcriptome

## Abstract

ADP-glucose pyrophosphorylase (AGPase) is a key rate-limiting enzyme involved in starch synthesis. APL1, an AGPase large subunit, plays an important role in the growth and development of grapes; however, its function in withstanding low temperature (LT) remains elusive. Hence, *VaAPL1* was cloned from *Vitis amurensis* (Zuoshan I), and its function was characterized. The gene was highly expressed in the phloem of *V. amurensis* during winter dormancy (0, −5, and − 10°C). Phylogenetic relationships demonstrated that VaAPL1 was closely genetic related to SlAPL1 (from *Solanum lycopersicum*), and clustered into I group. Further, *VaAPL1* was ectopically expressed in *Arabidopsis thaliana* (ecotype Columbia, Col) and tomato (“Micro-Tom” tomato) to characterize its function under LT. Compared with Col, the average survival rate of *VaAPL1*-overexpressing *A. thaliana* exceeded 75.47% after freezing treatment. Moreover, reactive oxygen species (ROS) content decreased in *VaAPL1*-overexpressing *A. thaliana* and tomato plants under LT stress. The activities of AGPase, and starch contents in *VaAPL1*-overexpressing *A. thaliana* were higher than in Col after LT stress. The contents of sucrose and glucose were accumulated in overexpressing plants compared with wild-type at 0 h and 24 h after LT stress. Transcriptome sequencing of overexpressing tomato plants revealed involvement in sugar metabolism and the hormone signal pathway, and Ca^2+^ signaling pathway-related genes were up-regulated. Hence, these results suggest that overexpression of *VaAPL1* not only ensured sufficient starch converting into soluble sugars to maintain cell osmotic potential and provided energy, but also indirectly activated signal pathways involved in LT to enhance plant tolerance.

## Introduction

Low temperature (LT) is detrimental to plants. It causes a series of cell dysfunctions, instability of the protein complex and RNA secondary structure, and inhibition of the metabolic activities of enzymes and other physiological processes (Chinnusamy et al., 2007). Changes in the composition and permeability of membrane lipids cause disturbances in membrane protein function, leading to wilting, yellowing, and even death of plants under LT (Zhu, 2016; Hoermiller et al., 2017). LT acclimation is a widely used approach in plant evolution. However, this process involves complex physiological and biochemical changes in plants, including gene expression, dynamic balance of reactive oxygen species (ROS), and formation of osmoregulatory substance. Thus, understanding the molecular regulatory mechanisms of plant stress response and resistance is of great significance for improving crop stress tolerance.

Starch metabolism provides a carbon skeleton for the synthesis of other substances and facilitates the continuous synthesis and export of sucrose (Dusenge et al., 2019). Starch is mainly synthesized during the daytime in mesophyll cells (primary photosynthetic tissue) and then remobilized at night (Lloyd and Kossmann, 2015). The initial step for starch biosynthesis is catalyzed by the rate-limiting enzyme ADP-glucose pyrophosphorylase (AGPase), which transforms glucose-1-phosphate and ATP into ADP-glucose and inorganic pyrophosphate (Meng et al., 2020). In higher plants, AGPase contains two large subunits (AGP-L) and two small subunits (AGP-S) which are encoded by two distinct genes (Salamone et al., 2000). The small subunit possesses catalytic and regulatory functions, while the large subunit is primarily responsible for the conformational regulation of AGPase (Zhou et al., 2016; Hou et al., 2019). A prior study reported a linear correlation between starch accumulation and the activity of AGPase during starch synthesis in plants, and some or even all of the starch synthesis was obstructed due to the inhibition of AGPase activity in wheat (Smidansky et al., 2002). Therefore, AGPase is considered to be a major enzyme controlling starch synthesis.

Six genes encode proteins homologous to AGPase in *A. thaliana*. Among these genes, *AtAPS1* and *AtAPS2* encode small subunits, and *AtAPL1*–*AtAPL4* encode large subunits (Crevillén et al., 2003). Abdellatif et al. (2011) determined that *AtAPS1*, *AtAPL1*, and *AtAPL2* are catalytically active, but *AtAPL3* and *AtAPL4* have lost their allosteric regulatory functions during evolution. Fritzius et al. (2001) demonstrated the ability of trehalose to induce the activity of AGPase in seedlings lacking the AGPase large subunit APL2 of the Arabidopsis mutant and to promote starch synthesis, however, this was not recorded for the *apl1* mutant. Four large subunit proteins of AGPase in rice, including OsAPL1, OsAPL2, OsAPL3, and OsAPL4, are synthesized in plastids (Takashi et al., 2005). In a previous study, the two subunits of AGPase in rice, *OsAPL1* and *OsAPS1*, were found to positively respond to nitrogen and phosphorus starvation (Meng et al., 2020). Both low nitrogen and low phosphorus levels triggered starch accumulation in the leaves of wild-type rice; however, this was not triggered in *apl1*, *aps1*, and *apl1 aps1* double mutants. Additionally, OsAPL1 and OsAPL3 were mutated, resulting in a decrease in AGPase activity by 23 and 1%, respectively (Rosti et al., 2007; Cook et al., 2012). Under LT and salt treatments, *MaAPL-2a* and *MaAPL-2c* were up-regulated in banana leaves. In grapevine leaves, AGPase activity was significantly induced by downy mildew (*Plasmopara viticola*) and led to abnormal starch accumulation (Gamm et al., 2011). Additionally, *SlAPL1* was up-regulated, resulting in starch accumulation in developing fruits of tomato (*Solanum lycopersicum*) under salinity stress. Drought stress inhibits AGPase activity, leading to a significant reduction in crop yield (Thitisaksakul et al., 2012). Further, heat stress reduced the activity of AGPase in wheat (Ulfat et al., 2021). All of these studies indicate that AGPsae plays an essential role in the regulation of plant growth and development, and responds to various stressors.

Grape is a widely cultivated fruit crop used for the production of table grapes, wine, juice, and dry raisins. However, the productivity and quality of grapes are easily affected by extreme freezing in winter and late frosts in spring. *Vitis amurensis* (a wild species) has been extensively explored because it can safely survive temperatures as low as ˗40°C (Xu et al., 2014). Numerous LT-response genes have been reported in *V. amurensis* (Zhang et al., 2019). Our transcriptome data showed that the *VaAPL1* gene from *V. amurensis* was significantly up-regulated in the phloem during winter dormancy periods, other isoforms did not changing trend (Liang et al., 2022). Moreover the *VaAPL1* contributes to LT tolerance in plants remains elusive. Therefore, the present study aimed to clarify whether *VaAPL1* is involved in sugar metabolism and if it regulates genes expression of antioxidation and cold signal transduction to improve LT tolerance in plants.

## Materials and Methods

### Plant Materials and Growth Conditions

Two-year-old *V. amurensis* (Zuoshan 1) was planted in the vineyards of the Gansu Agricultural University (103.69 ° E, 36.09 ° N). The plant had six main branches and no secondary shoots. We set five temperature phases to collect the phloem from annual branches according dormancy process as follows: the summer growth phase (air temperature was 28 ± 2°C), the early phase of cold hardening (air temperature was 5 ± 2°C), the middle phase of cold hardening (air temperature was 0 ± 2°C), the late phase of cold hardening (air temperature was −5 ± 2°C), and the deep dormancy phase (air temperature was −10 ± 2°C). At each phase of dormancy process, we randomly selected three plants as three replicates, and each plant was regarded as an independent replicate. The phloem from branches at the third to seventh internodes upward from the base was collected at 10 am. For the starch content in the phloem of *V. amurensis* branches in different temperature periods during winter dormancy, the cortex tissues of the branches were removed with a knife, and the phloem materials were carefully scraped to avoid collecting xylem. The phloem from branches at −5 ± 2°C during dormancy was performed total RNA extraction and *VaAPL1* cloning. *A. thaliana* ecotype Columbia (Col) was used as the wild-type and subsequent transformation material. The growth temperature and light intensity were 23°C/18°C (16-h-light/8-h-dark) and 300 μmol·m^−2^·s^−1^, respectively. Three-week-old Col and *VaAPL1*-overexpressing *A. thaliana* seedlings were subject to LT and freezing treatments, as described by Sun et al. (2019). For the LT treatment, 3-week-old seedlings of Col and *VaAPL1*-overexpressing *A. thaliana* were treated at 4°C for 24 h, and the plants growing under non-stress conditions were used as controls. Each experiment was performed in triplicates. For the survival rate treatment, Col and *VaAPL1*-overexpressing *A. thaliana* plants were acclimated at −1°C for 5 h and then cooled at a rate of 1°C·h^−1^ until −8°C for 3 h. The survival rate was determined 3 days after treatment. Sixteen seedlings of gene type were included and each experiment was performed in triplicates.

Tomato seeds (*S. lycopersicum* cv. “Micro-Tom”) were surface sterilized and sown on half-strength solid MS medium (containing 20 g·l^−1^ sucrose and 6.5 g·L^−1^ agar; pH 5.8) at 27°C in dark for 3 d. Then, the germinative seeds were maintained at 25°C/18°C (16-h-light/8-h-dark) under a light intensity of 100 μmol·m^−2^·s^−1^ to grow for 6–7 d. The WT and T_3_ generation overexpressing lines (OEs) were grown in a culture matrix (ratio of vermiculite to nutrition soil was 1:3). The LT treatment was given by gradient temperature-reduction method; the temperature was reduced from 25°C to 14°C at a rate of 2°C·h^−1^, and then from 14°C to 6°C at a rate of 1°C·h^−1^. Six-week-old WT and three OEs were subject to LT stress at 6°C after 24 h for histochemical staining, electrical conductivity determination, enzyme activities and carbohydrate content, transcriptome sequencing (controls were not subjected to any stress). Each transgenic line was consisted of thirty seedlings and identical results. Images were taken to record the phenotypes. Each physiological index contained three biological replicates and technical replicates.

### Determination of Starch Content

Starch content was determined using a commercial kit (Jiangsu Keming Biotechnology Institute, Suzhou, China) according to the manufacturer’s instructions. The sample weight for starch content detection was 0.2 g fresh plant materials. Finally, the absorbance of starch content was measured at 620 nm on UV–visible spectrophotometer (UV-1780, Shimadzu, Japan). Process of determination included three technical repetitions.

### RNA Isolation, Gene Cloning, and Homology Analysis

Total RNA from grape, *A. thaliana*, and tomato leaves was isolated using a commercial OMEGA plant RNA kit (Omega Bio-tek, Norcross, GA, United States) following the manufacturer’s protocol. First-strand cDNA was synthesized from 1 μg of RNA using the PrimeScript IV first strand cDNA Synthesis Mix kit (Takara Bio, Japan) according to the manufacturer’s instructions. Grape cDNA was used to amplify ORF (open reading frame) of the *VaAPL1*. The primers were as follows: forward primer, 5′-GACACCCATGGTAGATTCTTGCTGTGTGACCTTC-3′; reverse primer, 5′-GACACGGTCACCTTAGATAACTGTGCCATCCTTG-3′. The amplification product was detected *via* 1% agarose gel electrophoresis with 0.1% Goldview nucleic acid gel stain, and then inserted into the pMD20-T vector (Takara Bio, Japan) for sequencing verification (Supplementary Figure S1A). The sequence of *VaAPL1* was cloned into the *Nco* I and *BstE* II restriction sites of the vector pCAMBIA1301 to construct the *35S::VaAPL1* recombinant plasmid (Supplementary Figure S1B). Recombinant plasmids were checked for gene insertion sites using *Nco* I and *BstE* II digestion (Supplementary Figure S1C). The *VaAPL1*-pCAMBIA1301 recombinant plasmid was introduced into *Agrobacterium tumefaciens* strain GV3101 to acquire *VaAPL1*-overexpressing *A. thaliana* and tomato plants.

The APL protein sequences of *A. thaliana*, tomato, rice, peach, apple, and orange were retrieved from the *A. thaliana* data,^1^ tomato data,^2^ rice data,^3^ genome database for rosaceae,^4^ and orange data,^5^ respectively (Supplementary Table S1). An unrooted phylogenetic tree was created using MEGA 7.0 software based on the neighbor-joining method and 1000 bootstrap replicates. Colors were added using the online tool EvolView.^6^ The multiple sequence alignments of VaAPL1 and AtAPL proteins were performed using the ClustalX v.2.0 with default settings.

### Subcellular Localization

The PCR product, *VaAPL1* (without the termination codon) was then ligated into the pCAMBIA1300 vector and fused with GFP. The amplification primers were as follows: forward primer, 5′-GAGCTCGGTACCCGGGGAT CAATGGATTCTTGTTGTGTGACCTTCAA-3′ and reverse primer, 5′-CATGTCGACTCTAGAGGAT CCTTAGATAACTGTGCCATCCTTGATTG-3′. *35S*::*VaAPL1*-GFP and *35S*::*GFP* (control) plasmids were transferred into *A. tumefaciens* strain GV3101, and tobacco leaves were injected with these bacterial suspensions, which were cultivated in a chamber for 48 h. Protein subcellular localization was observed using a confocal laser scanning microscope (LSM 700, ZEISS, Germany).

### *Arabidopsis thaliana* and Tomato Transformation

*Arabidopsis thaliana* adopted the inflorescence transformation method to acquire T_0_ generation of *VaAPL1*-overexpressing plants as suggested by Zhao et al. (2018). *VaAPL1*-overexpressing *A. thaliana* plants were identified using MS solid medium (MS medium contain 20 g·l^−1^ sucrose, 6.5 g·l^−1^ agar, pH 5.8) containing 25 mg·l^−1^ hygromycin and PCR (Supplementary Figure S1D). Finally, the *VaAPL1*-overexpressing *A. thaliana* lines were used to generate T_3_ plants for subsequent LT stress analysis.

Tomato cotyledons were used as materials for *A. tumefaciens*-mediated leaf transformation as described by Yan et al. (2013) (Supplementary Figures S2A–D,F). Subsequently, PCR was performed to detect these transgenic plants using a pair of *VaAPL1* primers (Supplementary Figure S2G). The OEs were selected to produce T_3_ generation OEs for subsequent LT stress. The T_1_ generation OE seeds were collected and screened on 1/2 MS solid medium containing 15 mg·l^−1^ hygromycin. T_2_ generation was screened for homozygous plants, and seeds were harvested from individual lines to obtain T_3_ hygromycin-resistant OE seeds, which were used in the subsequent experiments. All primers were synthesized by Sangon Biotech Co., Ltd. (Shanghai, China), and are listed in Supplementary Table S2.

### Histochemical Staining and Electrical Conductivity Determination

The leaves of *A. thaliana* and tomato plants under normal growth and LT stress were stained with trypan blue, nitroblue tetrazolium (NBT), and 3, 3′-diaminobenzidine (DAB) to evaluate dead cells and the accumulation of O_2_^−^ and H_2_O_2_, respectively (Feng et al., 2013; Ma et al., 2018). Briefly, leaves of *A. thaliana* and tomato were immersed in boiling trypan blue solution for 5 min, followed by discoloration using 2.5 g·ml^−1^ chloral hydrate for 2 h. Similarly, the treated leaves were immersed in 10 mM potassium phosphate buffer with 0.1% NBT (pH 7.8) and vacuum infiltrated for 5 min, followed by incubation for 2 h at room temperature. Thereafter, the leaves were boiled in the NBT solution for 2 min. Tomato leaves were immersed in an aqueous solution of 1 mg·ml^−1^ DAB (pH 7.0), incubated in the dark for 8 h at room temperature, and then soaked in boiling water for 5 min. Stained *A. thaliana* leaves were decolorized with absolute ethanol for 2 h. Leaves dyed with NBT and DAB were decolorized with a solution (ethanol: lactic acid: glycerin, 3:1:1) until there was no chlorophyll. The relative electrical conductivity (REC) was determined using the methods described by Ma et al. (2018).

### Detection of Enzyme Activities and Carbohydrate Content

Activities of peroxidase (POD), superoxide dismutase (SOD), and catalase (CAT) in *A. thaliana* and tomato leaves were determined using a commercial ELISA kit (Yaji Biotechnology Institute, Shanghai, China), according to the manufacturer’s protocol. Fresh leaf samples (0.2 g) of *A. thaliana* and tomato were collected to measure AGPase and sucrose synthase (SuSy) activities using a commercial kit (Jiangsu Keming Biotechnology Institute, Jiangsu, China) according to the manufacturer’s protocol. The sucrose and glucose contents were determined as previously described (Smith and Zeeman, 2006). Fructose content was measured using a commercial kit (Jiangsu Keming Biotechnology Institute, Jiangsu, China) according to the manufacturer’s protocol.

### Transcriptome Sequencing of OEs

RNA degradation, integrity, and purity of WT and OEs were estimated using 1% agarose gel electrophoresis, Agilent 2,100 Bioanalyzer (Agilent Technologies, CA, United States), and NanoDrop spectrophotometer (NanoDrop Technologies, CA, United States), respectively. RNA was quantified using a Qubit^™^ 4 fluorometer (Invitrogen, CA, United States). Total RNA (3 μg) from each sample was used to construct a sequencing library. Transcriptome sequencing of WT and OE leaves at 6°C for 24 h was performed by Biomarker Technologies Co., Ltd. (Beijing, China). Subsequently, the NovaSeq 6,000 sequencing system was used for sequencing. Ultimately, clean data were obtained by removing reads containing adapter and low quality reads (reads with unrecognizable “N” bases and those with more than 10% wrong sequencing bases) from raw data. The clean data were aligned to the tomato genome (https://solgenomics.net/) using TopHat v2.0.12. Differential expression analysis of the WT and OE leaves was performed using the DESeq R package (1.18.0). The threshold parameters of differentially expressed genes (DEGs) were as follows: false discovery rate (FDR) < 0.05 and fold change (|log2FC|) ≥ 2. Significance for KEGG pathway was determined at *p* ≤ 0.05. Transcriptome sequencing samples consisted of WT and three separate OEs.

### qRT-PCR Analysis

Starch- and sucrose-related gene expression in *A. thaliana* and tomato plants was analyzed using qRT-PCR before and after LT treatments. qRT-PCR was performed as previously described by Bennett et al. (2015) using TB Green^®^ Premix Ex Taq^™^ II (Takara Bio, Japan), according to the manufacturer’s instructions. The reaction mixture (20 μl) consisted of 1 μl of cDNA (100 ng·μl^−1^), 10 μl of TB Green^®^ Premix Ex Taq^™^ II, 2 μl of gene-specific primers (1 μl each of forward and reverse primers), and 7 μl of RNase free water. The program was initiated with a preliminary step of 1 min at 95°C, followed by 40 cycles at 95°C for 10 s, 55°C for 30 s, and 72°C for 20 s using a Light Cycler^®^96 Real-Time PCR system (Roche, Switzerland). The reference genes for *A. thaliana* and tomato were *AtACT* (AT3G46520) and *SlACT* (NM_001330119), respectively. Gene expression levels were calculated using the 2^−ΔΔCt^ method (Livak and Schmittgen, 2001). qRT-PCR experiments were performed in three biological replicates.

### Statistical Analysis

Microsoft Excel 2010 software was used for data collation. All experimental data are showed as the mean ± standard deviation (SD) of three independent biological replicates. Data were analyzed by one-way ANOVA using the SPSS software (version 22.0; SPSS Inc., Chicago, IL, United States). Different letters indicate significantly different means (*p* < 0.05, Duncan’s multiple range tests). Figures were drawn using Origin 9.0 (Microcal Software) software.

## Results

### Difference of Starch Content in Phloem, Phylogenetic Relationship of APLs, and Multiple Sequence Alignment

**Figure 1:**
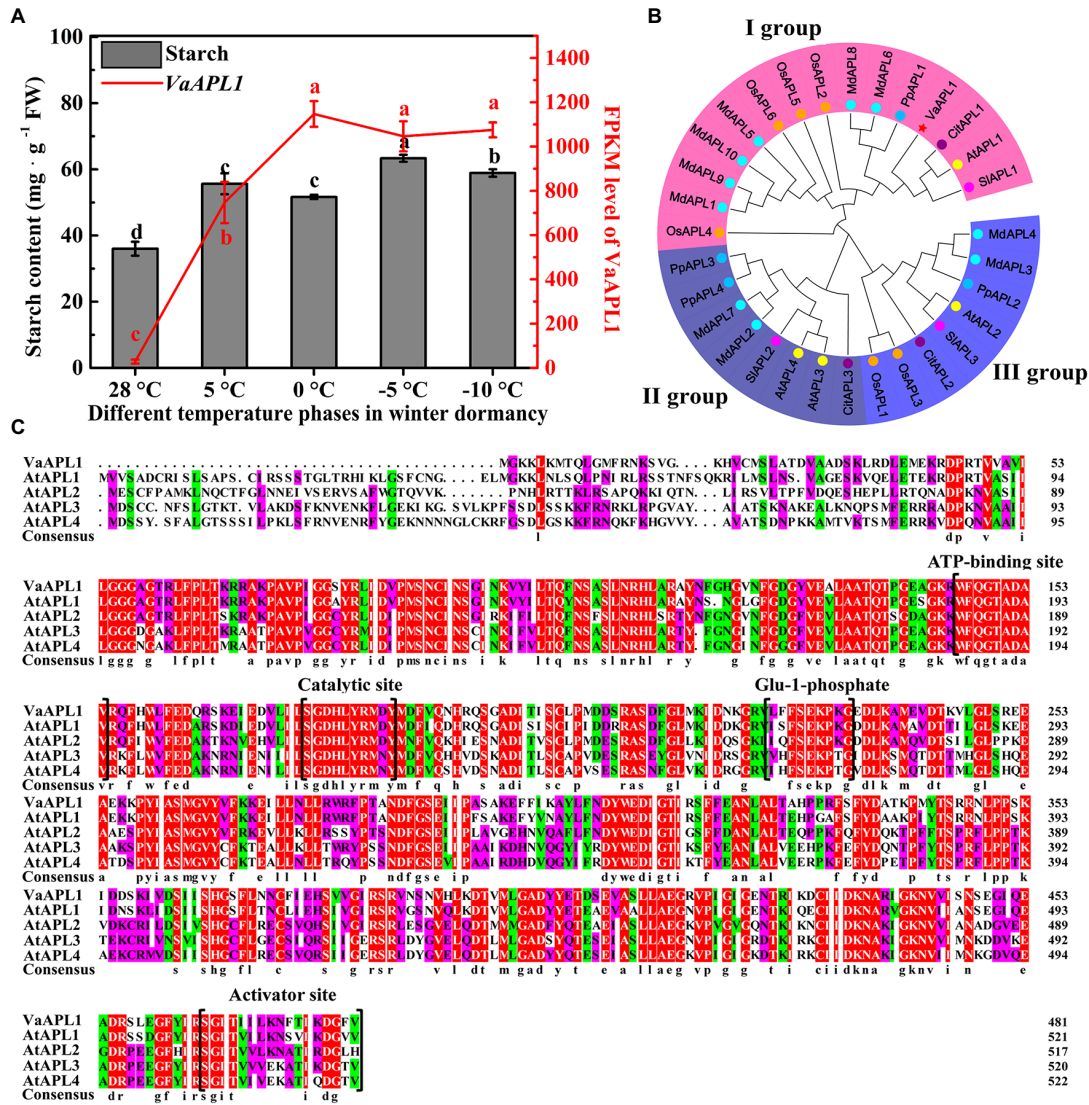
Starch content and *VaAPL1* expression in branches phloem of *Vitis amurensis* under different temperature treatments, APL protein phylogenetic relationship, and multiple sequence alignment of VaAPL1 and AtAPL. **(A)** Change of starch content and *VaAPL1* expression levels in the phloem of *V. amurensis* at different temperature phases in winter dormancy. **(B)** Phylogenetic relationship of APL1 proteins from six species. These species included *A. thaliana*, tomato, rice, peach, apple, and orange. Different colored fan-shaped areas represent different subfamilies. Circles of the same color represent the same species. Red star represents VaAPL1. **(C)** Multiple sequence alignment between VaAPL1 and AtAPL proteins.

Starch content exhibited an initial increasing trend followed by a decrease as temperature declined in the phloem of *V. amurensis* branches. The highest peak appeared at 5°C, and the lowest content at 28°C (Figure 1A, gray histogram). Analysis of the previous transcriptome data revealed that the expression of *VaAPL1* significantly increased with decreasing temperature, and the highest transcript accumulation was at 0°C (Figure 1A, red broken-line). These results suggested that starch can accumulate with decreasing temperature. Additionally, APL protein members from six species were retrieved—apple (MdAPL1–MdAPL10), *A. thaliana* (AtAPL1–AtAPL4), tomato (SlAPL1–SlAPL3), rice (OsAPL1–OsAPL6), peach (PpAPL1–PpAPL4), and orange (CitAPL1–CitAPL3; Figure 1B; Supplementary Table S1). These APL1 proteins were clustered into three groups. VaAPL1 clustered into group I and was most closely related to SlAPL1, displaying a sequence similarity of 66.60%. These results indicate the VaAPL1 has a close relationship with SlAPL1. Furthermore, the VaAPL1 contained four key domains of: ATP-binding site, catalytic site, Glu-1-phosphate catalytic site, and activator site (Figure 1C).

### *VaAPL1* Subcellular Localization and Evaluation of Antioxidant Enzymes in *VaAPL1*-Overexpressing *Arabidopsis thaliana* Under LT

**Figure 2:**
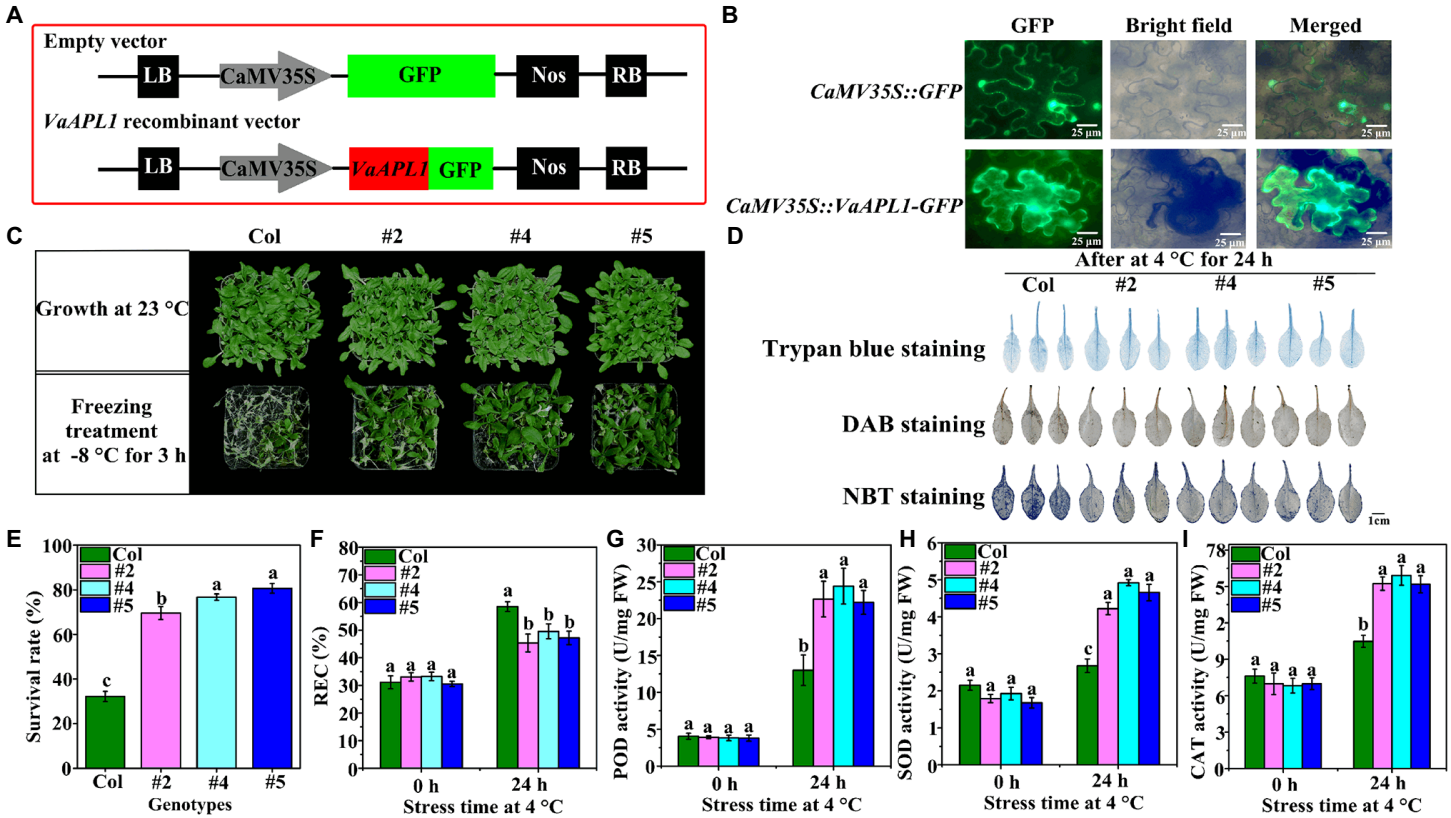
Subcellular localization, survival rate, and changes in physiology and biochemistry of *VaAPL1*-overexpressing *A. thaliana* plants. **(A)** The vector maps of subcellular localization. **(B)** Subcellular localization of *VaAPL1* in leaf epidermal cells of tobacco. **(C)** Phenotypes of the Col and *VaAPL1*-overexpressing *A. thaliana* plants (three individual transgenic lines were labeled #2, #4, and #5) after freezing treatments (−1°C for 2 h, and then cooled at a rate of 1°C·h^−1^ until −8°C for 3 h). **(D)** The leaves of Col and *VaAPL1*-overexpressing *A. thaliana* plants were stained using DAB, NBT, and trypan blue. **(E)** Survival rate of Col and *VaAPL1*-overexpressing *A. thaliana* plants after freezing treatment. **(F)** The change of relative electrolyte leakage (REC) in Col and *VaAPL1*-overexpressing *A. thaliana* plants observed at 4°C after 0 h and 24 h. **(G**–**I)** The changes in POD, SOD, and CAT activities in Col and *VaAPL1*-overexpressing *A. thaliana* plants at 4°C after 0 h and 24 h.

The subcellular localization of *VaAPL1* was detected by its transient expression in tobacco using an empty vector as a control (Figure 2A). Overexpression of VaAPL1 was driven using the 35S promoter. GFP fluorescence of the empty vector was observed in the cell membrane and nucleus (Figure 2B). However, the GFP fluorescence of the fusion *VaAPL1* was mainly expressed in the cytoplasm. Overall, these results indicate that VaAPL1 functions in the nucleus and cytoplasm.

To further investigate the role of *VaAPL1* under LT stress, five T_3_ homozygous *VaAPL1*-overexpressing *A. thaliana* lines were generated using the floral dip method, among which we selected three independent plants (three independent transgenic lines were labeled #2, #4, and #5) for subsequent experiments (Supplementary Figure S1D). The results showed that *VaAPL1*-overexpressing *A. thaliana* plants had a higher average survival rate (>75.47%) compared with Col (32.41%) after freezing treatment (Figures 2C,E). The staining results showed that the leaf color of Col plants was deeper than that of *VaAPL1*-overexpressing *A. thaliana* plants after 24 h of stress at 4°C (Figure 2D). The relative electrolyte leakage (REC) showed that there was no significant difference between Col and *VaAPL1*-overexpressing *A. thaliana* plants at 0 h; however, after 24 h at 4°C, the REC of *VaAPL1*-overexpressing *A. thaliana* plants was remarkably lower than that of Col (Figure 2F). Under normal conditions, the POD activity was not significantly different between Col and *VaAPL1*-overexpressing *A. thaliana* plants; however, after LT treatment, the POD activity of the overexpressing plants was significantly higher than that of Col (Figure 2G). SOD activity showed no significant difference between Col and *VaAPL1*-overexpressing *A. thaliana* plants at 0 h (Figure 2H); however, after 24 h at 4°C, the SOD activity in the leaves of *VaAPL1*-overexpressing *A. thaliana* plants was significantly higher than that of Col. The trend of CAT activity was consistent with POD and SOD at 0 h and 24 h (Figure 2I). These results suggest that *VaAPL1* overexpression increases LT tolerance in *A. thaliana*.

### Activity of AGPase and SuSy and Carbohydrate Contents in *Arabidopsis thaliana*

**Figure 3:**
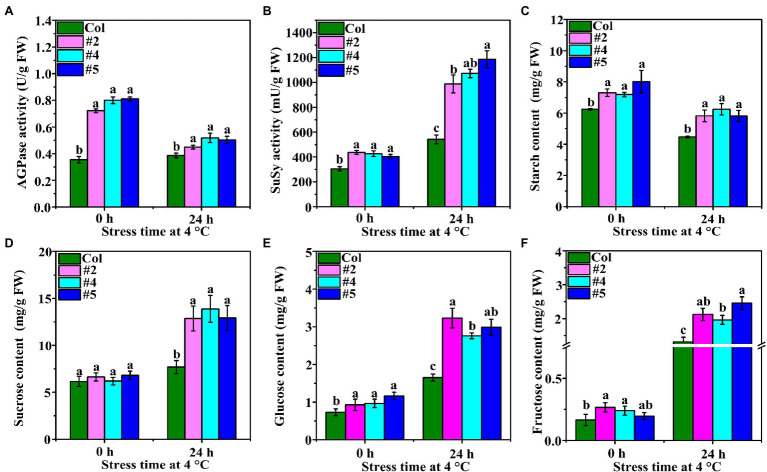
Changes in AGPase and SuSy enzyme activities and starch and soluble content. **(A,B)** The changes in AGPase and SuSy activities in Col and *VaAPL1*-overexpressing *A. thaliana* plants at 4°C after 0 h and 24 h. **(C)** The changes in starch content in Col and *VaAPL1*-overexpressing *A. thaliana* plants at 4°C after 0 h and 24 h. **(D–F)** The changes in sucrose, glucose and fructose content in Col and *VaAPL1*-overexpressing *A. thaliana* plants at 4°C after 0 h and 24 h.

The activity of AGPase in *VaAPL1*-overexpressing *A. thaliana* plants was significantly higher than that of Col plants at 0 h, and the same trend was observed after LT stress for 24 h (Figure 3A). SuSy activity was higher in *VaAPL1*-overexpressing *A. thaliana* plants than in Col plants after LT stress for 24 h (Figure 3B). The starch content in the leaves of *VaAPL1*-overexpressing *A. thaliana* plants was higher than that of Col plants under 0 h and LT stress for 24 h (Figure 3C). In addition, the starch contents in Col and *VaAPL1*-overexpressing *A. thaliana* plants were significantly lower than those under growth conditions. The sucrose content was not significantly different between Col and *VaAPL1*-overexpressing *A. thaliana* plants at 0 h; however, after LT stress, the OEs showed higher sucrose content than Col plants (Figure 3D). Glucose contents in leaves of *VaAPLI*-overexpressing *A. thaliana* were significantly higher than that of Col at 0 h. After LT stress, glucose content increased in both *VaAPLI*-overexpressing *A. thaliana* plants and Col, and transgenic lines were significantly higher than Col (Figure 3E). Fructose content was significantly higher than that of Col in *VaAPLI*-overexpressing *A. thaliana* (#2 and #4) leaves at 0 h (Figure 3E). After LT stress, fructose content was significantly increased in Col and *VaAPLI*-overexpressing, and transgenic lines were significantly higher than Col. These results suggest that *VaAPL1* overexpression provided sufficient starch to convert into soluble sugars, modulate osmotic potential and improve LT tolerance in *VaAPLI*-overexpressing *A. thaliana*.

### Expression of Starch Hydrolysis-Related Genes in *Arabidopsis thaliana* Under LT Stress

**Figure 4:**
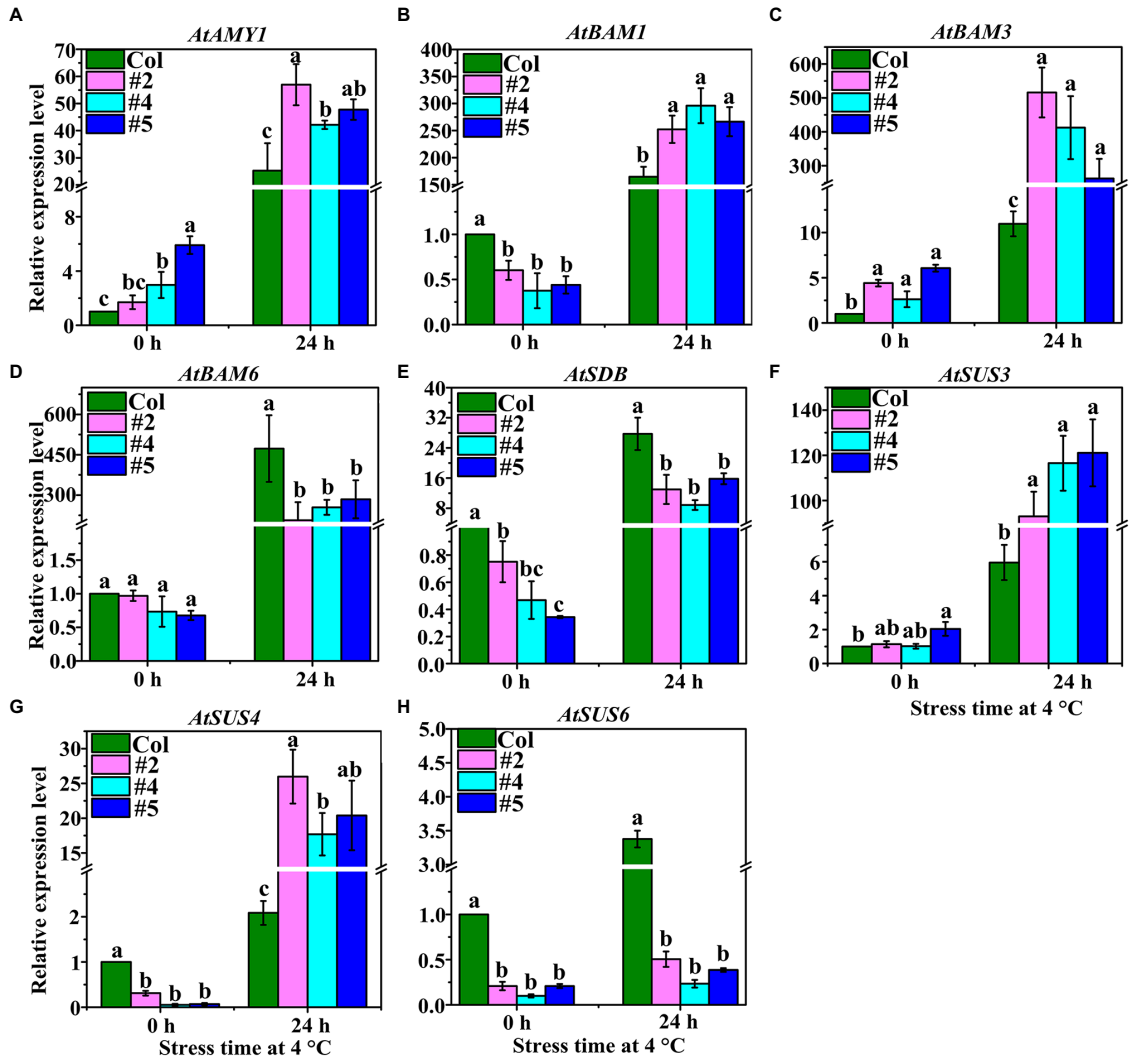
Starch and sucrose metabolism-related genes were analyzed by qRT-PCR in Col and *VaAPL1*-overexpressing *A. thaliana* plants at 4°C after 0 h and 24 h. These genes include: **(A)** α-amylase (*AtAMY1*); **(B–D)** β-amylase (*AtBAM1*, *AtBAM3*, and *AtBAM6*); **(E)** starch debranching enzyme (*AtSDB1*); **(F–H)** SuSy (*AtSUS3*, *AtSUS4*, and *AtSUS6*).

We used qRT-PCR to determine the expression levels of an α-amylase gene (*AtAMY1*), three β-amylase genes (*AtBAM1*, *AtBAM3*, and *AtBAM6*), one starch debranching enzyme gene (*AtSDB*), and three SuSy genes (*AtSUS3*, *AtSUS4*, and *AtSUS6*; Figure 4). The results showed that the expression of *AtAMY1* in *VaAPL1*-overexpressing *A. thaliana* plants (#4 and #5) was significantly higher than that in Col plants at 4°C after 0 h and 24 h (Figure 4A). Moreover, *AtAMY1* was significantly up-regulated in Col and *VaAPL1*-overexpressing *A. thaliana* plants after LT stress. The expressions of *AtBAM1* and *AtBAM3* were significantly up-regulated in *VaAPL1*-overexpressing *A. thaliana* plants after 24 h at 4°C, and their expression levels were higher than those of Col (Figures 4B,C). The expression level of *AtBAM6* in *VaAPL1*-overexpressing *A. thaliana* plants was significantly lower than in Col plants at 4°C after 24 h, however, it was up-regulated in both Col and *VaAPL1*-overexpressing plants at 4°C after 24 h (Figure 4D).

Interestingly, *AtSDB* was up-regulated in both Col and *VaAPL1*-overexpressing plants at 4°C after 24 h, however, its expression level in transgenic plants was significantly lower than that in Col (Figure 4E). The expressions of two SuSy genes, *AtSUS3* and *AtSUS4*, were significantly up-regulated in both Col and *VaAPL1*-overexpressing plants at 4°C after 24 h, and their expression levels in transgenic plants were significantly higher than those in Col (Figures 4F,G). However, *AtSUS6* was highly expressed in Col compared to *VaAPL1*-overexpressing plants (Figure 4H). These results indicate that *VaAPL1* overexpression increased the expression of starch and sucrose hydrolysis-related genes.

### Changes in Phenotype, Antioxidant Enzymes Activities and Carbohydrate Contents in OEs

**Figure 5:**
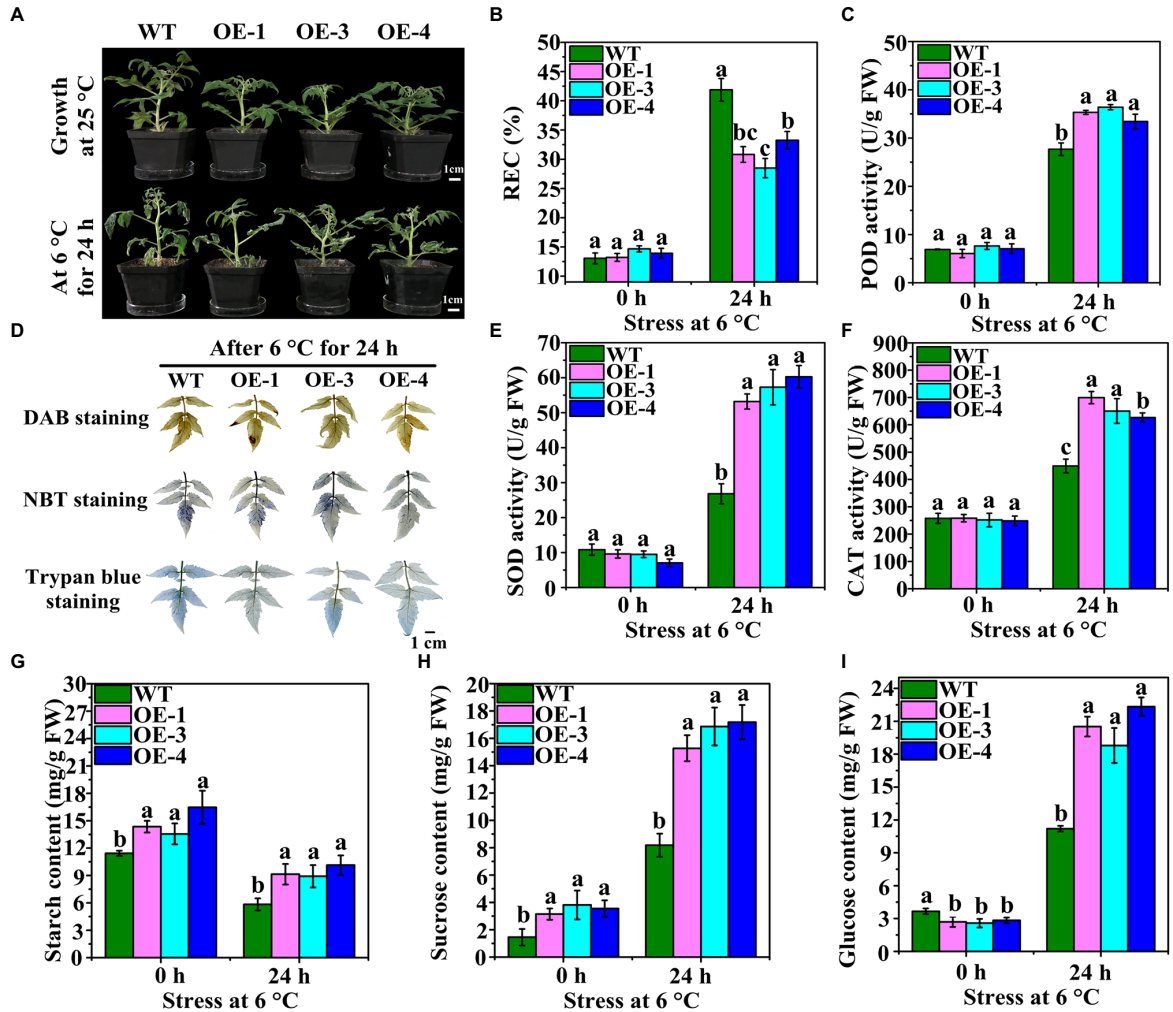
Phenotypes and antioxidant enzymes of OEs under LT treatment. **(A)** Phenotypes of OEs (OE-1, OE-3, and OE-4) and wild-type (WT) at 25°C and under LT stress at 6°C after 24 h. **(B)** DAB, NBT, and trypan blue staining of WT and OE leaves at 6°C for 24 h. **(C)** REC change in WT and OEs were subjected to LT treatment at 6°C for 0 h and 24 h. **(D**–**F)** The changes in POD, SOD, and CAT activities in WT and OEs subjected to LT stress at 6°C after 0 h and 24 h. **(G**–**I)** The changes in starch, sucrose, and glucose contents in WT and OEs subjected to LT stress at 6°C after 0 h and 24 h.

The germination rate of T_3_ seeds of the WT was found to be approximately 95%, but that of OEs was only 48.56–58.28% (Supplementary Figure S3A). The height of the OEs was significantly lower than that of the WT plants (Figure 5A; Supplementary Figure S3B). Moreover, OE leaves were less wilted less than WT leaves at 6°C after 24 h (Figure 5A). Cell damage and accumulation of H_2_O_2_ and O_2_^−^ in WT and OEs grown at 25°C and after LT stress were analyzed by staining. Staining with DAB, NBT, and trypan blue showed that the leaf color of WT was deeper than that of OEs after LT stress (Figure 5B). The REC of leaves did not differ between WT and OEs at 0 h (Figure 5C). However, after LT stress, the REC of OEs was significantly lower than that of WT plants. The antioxidant enzymes activities were also measured. OEs showed higher POD, SOD, and CAT activities than WT plants at 6°C after 24 h (Figures 5D–F). These results suggest that OEs have strong ROS scavenging potential.

Starch content significantly differed between WT and OEs at 25°C and after LT stress (Figure 5G). The starch content in the OEs was significantly higher than in the WT prior to LT stress. After LT stress, starch content decreased in both OEs and WT, however, it was significantly higher in OEs than in WT. At 6°C and 0 h, the sucrose content in the OEs was significantly higher than the WT plants (Figure 5H), and this trend was maintained even after LT stress. The glucose content of the OE leaves was lower than that of the WT at 6°C and 0 h; however, it was higher than the WT at 6°C after 24 h (Figure 5I). These results suggest that overexpression of *VaAPL1* not only promoted starch accumulation, but also increased sucrose and glucose content under LT stress.

### Expression of Key Genes Related to Starch Hydrolysis and Sucrose Synthesis in OEs

**Figure 6:**
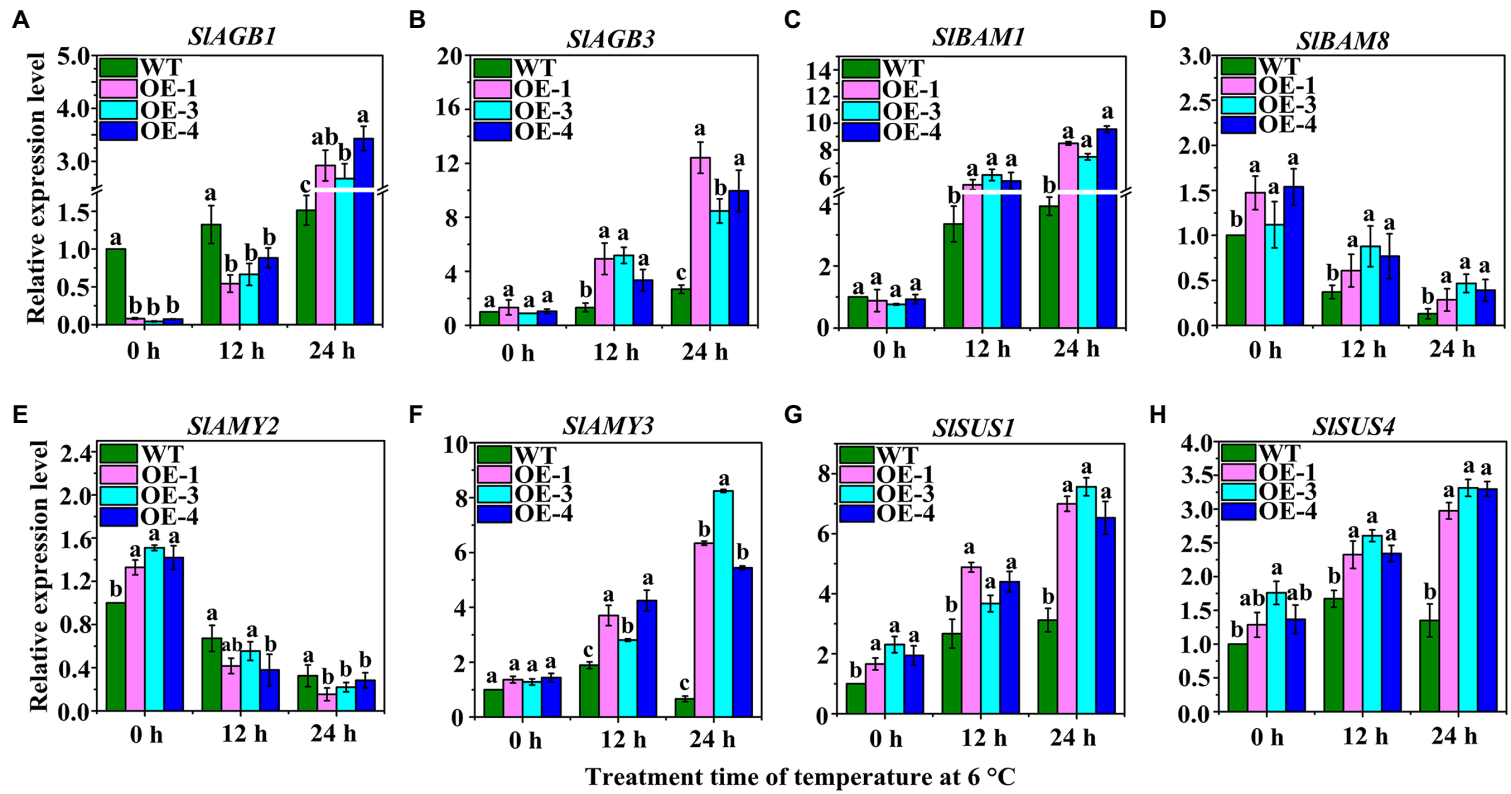
The expression levels of sucrose synthesis- and starch hydrolysis-related genes in OEs under LT treatment. These genes include: **(A,B)** *SlAGB1* and *SlAGB3*; **(C,D)**, β-amylase genes (*SlBAM1* and *SlBAM8*); **(E,F)** α-amylase genes: *SlAMY2* and *SlAMY3*; **(G,H)** SuSy genes: *SlSUS4* and *SlSUS6*.

The expression of key genes involved in starch hydrolysis and SuSy was analyzed by qRT-PCR (Figure 6). The results showed that 1, 4-alpha-glucan-branching enzymes (*SlAGB1* and *SlAGB3*) were significantly up-regulated in OEs at 6°C after 12 h and 24 h of LT stress (Figures 6A,B). Interestingly, the expression of *SlAGB1* was significantly down-regulated in OEs compared with WT after 0 h and 12 h under LT stress, but was up-regulated after 24 h (Figure 6A). The expression level of *SlAGB3* was continuously up-regulated over time but was highly expressed in OEs (Figure 6B). The expression of *SlBAM1* was up-regulated in WT and OEs under LT stress, and was also significantly expressed in OEs compared to WT (Figure 6C). At different time points during LT stress, the expression levels of *SlBAM8* in OEs was always higher than that in WT, but the overall expression decreased over time (Figure 6D). *SlAMY2* was highly expressed in OEs compared with WT at 0 h, however, it was gradually down-regulated under LT stress LT stress over time (Figure 6E). The expression level of *SlAMY3* was gradually up-regulated in OEs under LT stress over time; however, it was gradually down-regulated in the WT (Figure 6F). Two SuSy genes, *SlSUS1* and *SlSUS4*, were highly expressed in OEs under LT stress over time, and their expression levels gradually increased (Figures 6G,H). These results suggest that sufficient starch was hydrolyzed into soluble sugars to enhance the LT tolerance of OEs.

### Transcriptome Analyses of DEGs in OEs

**Figure 7:**
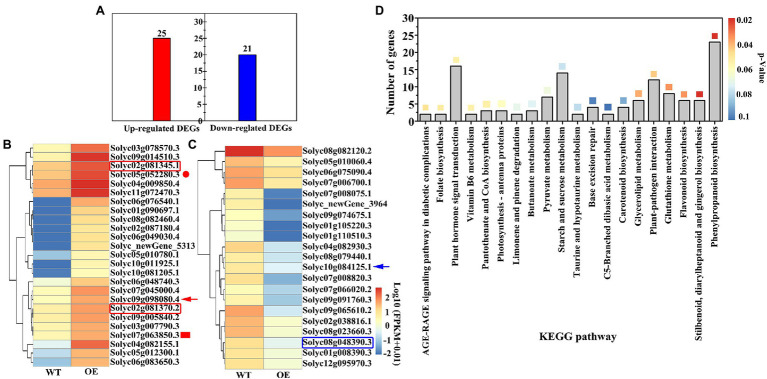
Analysis of differentially expressed genes (DEGs) and KEGG annotation information. **(A)** The amount of DEGs in OEs after LT stress for 24 h. **(B)** Cluster and heat map analysis of total up-regulated DEGs. **(C)** Cluster and heat map analysis of total down-regulated DEGs. **(D)** The top 20 KEGG pathways were annotated in OEs after LT stress for 24 h. The different color boxes represent the enrichment significance level (*p* < 0.05).

The average total read amount of transcripts per sample was 228,653,697; the mapped reads ranged from 62.88 to 86.32%, and the unique mapped reads ranged from 56.61 to 76.96%, indicating that the sequencing data can be used for subsequent analysis (Supplementary Table S3). A total of 46 DEGs were screened using the DEG threshold parameters, including 25 up-regulated and 21 down-regulated DEGs (Figure 7A; Supplementary Table S4). DEG annotation results showed that the two peroxisomal genes (*Solyc02g081345.1*, *Solyc02g081370.2*, red box) were up-regulated by 6.71- and 6.17-fold in OEs, peroxidase 52 (*Prx52*, *Solyc05g052280.3*, red circle) gene was up-regulated by 6.76-fold, and the auxin-related gene, indole-3-acetic acid-amido synthetase GH3.6 (*Solyc07g063850.3*, red fill box), was up-regulated by 6.55-fold (Figure 7B). Specifically, the UDP-glycosyltransferase (*Solyc09g098080.4*, red arrow) gene was up-regulated by 9.22-fold. However, the UDP-glucuronate 4-epimerase 2 (*Solyc08g048390.3*, blue box) gene was down-regulated by 19.07-fold (Figure 7C). Interestingly, the abscisic acid receptor PYL2 (*Solyc10g084125.1*, blue arrow) gene was down-regulated 19.02-fold. Analysis of the top 20 KEGG pathways revealed that the phenylpropanoid biosynthesis pathway was the most significant and annotated 23 DEGs, followed by stilbenoid, diarylheptanoid, and gingerol biosynthesis pathway including 6 DEGs (Figure 7D). However, 14 DEGs were annotated for starch and sucrose metabolic pathways. These results indicate that *VaAPL1* overexpression indirectly up-regulated the expression of antioxidant enzymes and UDP-glycosyltransferase genes, and enhanced LT tolerance of OEs.

### DEGs of Sugar Metabolism and Hormone Signaling Pathway in OEs

**Figure 8:**
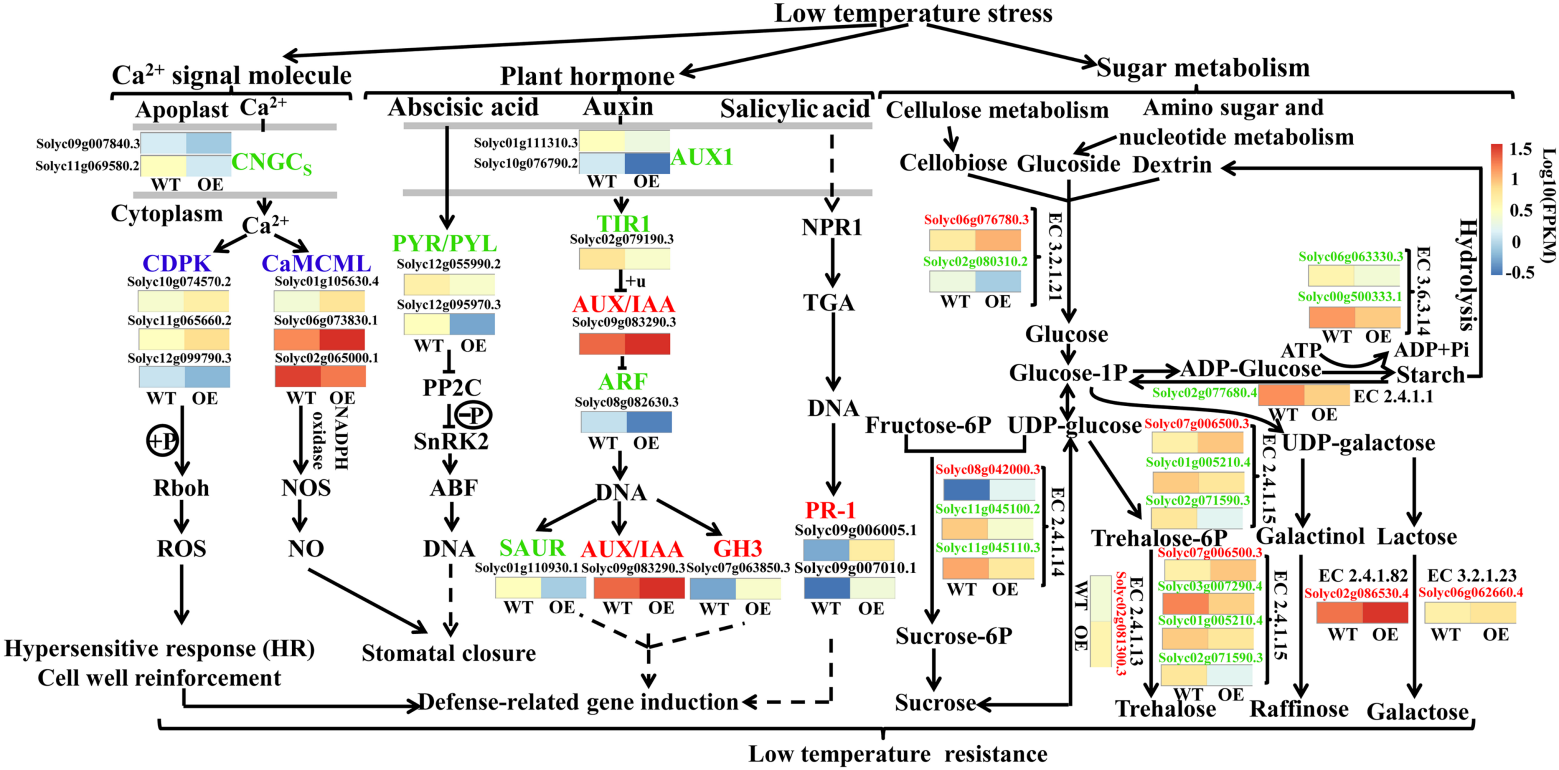
Involvement of other genes in the LT regulatory network in OEs. This regulatory network mainly includes sucrose metabolism, plant hormones, and Ca^2+^ signal regulation pathways. Blue color indicates that a protein or enzyme was annotated as both up- and down-regulated genes. Red color indicates that it was only annotated as up-regulated genes. Green color indicates that it was only annotated as down-regulated genes.

Overexpression of *VaAPL1* changed numerous aspects of hormone signaling and metabolism in OEs subjected to LT stress, such as “Plant hormone signal transduction” “MAPK signaling pathway” and sugar metabolism (Figure 8). In the Ca^2+^ signaling pathway, LT down-regulated two genes of cyclic nucleotide-gated channels (CNGCs, *Solyc09g007840.3*, *Solyc11g069580.2*) and up-regulated downstream genes, calcium-dependent protein kinase (CDPK, *Solyc10g074570.2*, *Solyc11g065660.2*) and calmodulin (CaMCML, *Solyc01g105630.4*, *Solyc06g073830.1*), in OEs. In the hormone pathway, the abscisic acid receptor PYR/PYL (*Solyc12g055990.2*, *Solyc12g095970.3*) was down-regulated in abscisic acid pathway; the auxin influx carrier (*Solyc01g111310.3*, *Solyc10g076790.2*) genes were down-regulated in the auxin pathway, and a gene of transport inhibitor response 1 (*Solyc02g079190.3*) was down-regulated. Down-regulation of these genes resulted in the up-regulation of auxin-responsive proteins IAA (*Solyc09g083290.3*) and GH3 (*Solyc07g063850.3*). Moreover, pathogenesis-related protein 1 (*Solyc09g006005.1*, *Solyc09g007010.1*) genes were up-regulated in the salicylic acid pathway. These genes can induce the expression of defense-related genes in OEs under LT stress. *VaAPL1* overexpression also affected changes in glucose metabolism-related genes. *VaAPL1* overexpression increased the expression of genes involved in sucrose (*Solyc02g081300.3*), trehalose (*Slyc07g006500.3*), raffinose (*Solyc02g086530.4*), and galactose (*Solyc06g062660.4*) synthesis after LT stress. These sugars can regulate the osmotic potential of plant cells and improve their LT resistance.

## Discussion

AGPase is a crucial rate-limiting enzyme in starch biosynthetic pathways and has been reported to possess important biological functions in response to environmental stress in plants (Mugford et al., 2014). Starch is an important nonstructural carbohydrate in plants response to abiotic stresses. Jiang et al. (2015) reported that the strong cold-tolerance grape xylem was filled with large quantities of starch compared to the weak grape varieties. The present study also showed that starch content gradually increased in the phloem of *V. amurensis* in the early phases of winter dormancy (Figure 1A), correlating to a study by Jiang et al. (2015). This suggests that sufficient starch degrades into more soluble sugars to provide energy and stabilize osmotic potential at LT. Georgelis et al. (2007) analyzed the evolutionary relationship between APL proteins in the angiosperms, demonstrating that these proteins were clustered into five groups. AtAPL3 and AtAPL4 were clustered into the same group. Our results showed that the APL proteins from the six species were clustered into three groups, and the AtAPL3 and AtAPL4 were clustered into II group (Figure 1B). Georgelis et al. (2007) also reported that SlAPL1 and AtAPL1 were not in the same group, however, our results showed that they were clustered together in I group and were closely related to VaAPL1. This difference is likely because of APL proteins of other species that were introduced for analysis. Analysis of AGPase large subunit gene families in bananas revealed that *MaAPL-2a* and *MaAPL-2c* were moderately up-regulated under LT and salt treatments (Miao et al., 2017). We also cloned a *VaAPL1*, which was up-regulated in the phloem of *V. amurensis* during the winter dormancy (Figure 1A). These results suggested that the *APL* genes from different plants also responded to abiotic stress. Additionally, the present work showed that the subcellular localization of *VaAPL1* is mainly in cytoplasm, and little in the nucleus. However, the result was different with Lee et al. (2007) and Meng et al. (2020). This may be caused by the short expression time of *VaAPL1* in tobacco mesophyll cells observed in this study (Jia et al., 2022; Ma et al., 2022). Our results indicated that overexpression of *VaAPL1* resulted in a decrease in REC in *VaAPL1*-overexpressing *A. thaliana* plants and OEs after LT stress (Figures 2F, 5B). This suggests that the integrity of the cell membrane was protected, thus laying the foundation for plant survival. In addition, freezing experiments showed that overexpression of *VaAPL1* increased the survival rate of *VaAPL1*-overexpressing *A. thaliana* plants (Figures 2C,E). After 24 h at 4°C, the color of the leaves of *VaAPL1*-overexpressing *A. thaliana* plants and staining of OEs with DAB, NBT, and trypan blue were slightly lower than that of the wild-type (Figures 2D, 5D). These results suggest that *VaAPL1* overexpression reduces the degree of ROS damage to cells and improves LT resistance in *VaAPL1*-overexpressing *A. thaliana* plants and OEs.

Plant tissues commonly face increasing accumulation of ROS when exposed to abiotic stress. ROS mainly include singlet oxygen (^1^O_2_), superoxide radical (O_2_^−^), hydrogen peroxide (H_2_O_2_), and hydroxyl radical (OH^−^; Rodrigo-Moreno et al., 2013). High accumulation of ROS destroys redox homeostasis, leads to cell death, and reduces biomass (Czarnocka and Karpiński, 2018). Therefore, excessive ROS must be scavenged. Antioxidant enzymes include POD, SOD, CAT, ascorbate peroxidase (APX), glutathione reductase (GR), and glutathione peroxidase (GPx). A dynamic equilibrium exists between ROS production and elimination in plants under environmental stress. ROS are scavenged not only through enzymatic defense systems but also by antioxidants such as ascorbate (AsA), glutathione (GSH), carotenoids, flavonoids, and proline (Czarnocka and Karpiński, 2018). Bolouri-Moghaddam et al. (2010) proposed the concept of sugar as an antioxidant, and it is becoming increasingly evident that sugars can also act as ROS scavengers in plants (Wolfe and Bryant, 1999; Morelli et al., 2003). Kathleen et al. (2008) reported that ensuring the normal function of AGPase can reduce the damage caused by ROS and stress in AGPase-deficient pea. Our results demonstrated that the activities of POD, SOD, and CAT in *VaAPL1*-overexpressing *A. thaliana* plants and OEs were significantly higher than those in wild-type plants under LT stress (Figures 2G–I, 5C,E,F). Furthermore, this result was consistent with the leaf color obtained by DAB and NBT staining in *VaAPL1*-overexpressing *A. thaliana* plants. This suggests that *VaAPL1* overexpression may indirectly contribute to scavenging excess ROS by increasing the activity of antioxidant enzymes.

Terrestrial plants must overcome the adverse effects of LT conditions to the greatest extent possible (Maruyama et al., 2014). However, the carbohydrate metabolism can be rapidly modulated in response to LT stress. Soluble sugars (including sucrose, glucose, fructose, maltose, raffinose, and trehalose) play an important role in acting as osmotic protectants as well as maintaining the biochemical function of the cell membrane *via* interaction with the lipid bilayer when faced with LT stress in plants (Anna et al., 2016; Fareen et al., 2016). A *PbrBAM3*-overexpressing *A. thaliana* exhibits better cold tolerance due to the high soluble sugar accumulation (Zhao et al., 2019). In addition, the accumulation of sugars in different plants significantly increased their response to various environmental stressors (Keunen et al., 2013). Plants can sense LT through sugar signals to ensure gene expression is altered to improve LT tolerance (Dahiya et al., 2017). Sucrose, a sugar signaling molecule, can regulate AGPase activity and control the change in starch content under LT conditions (Wiberley-Bradford et al., 2016). Moreover, α-amylase and β-amylase genes, encoding starch hydrolysis-related enzymes, were significantly up-regulated under LT or dehydration stress in rice, and soluble sugar content also increased significantly, indicating that sufficient starch contributes to regulation of cell osmotic potential by soluble sugars and improves plant tolerance (Maruyama et al., 2014). Akihiro et al. (2005) performed northern blot analysis with gene-specific probes, and their results showed that transcripts of AGPase large subunit, *OsAPL3*, significantly accumulated in response to sucrose and ABA concentration levels. In addition, the starch content of the cultured cells increased. Pérez et al. (2018) created two types of rice mutants with non-functional AGPase using CRISPR/Cas9 to illustrate that *OsAPS2b* and *OsAPL2* contribute to AGPase activity. These results indicated that starch depletion and soluble sugar levels in the leaves of both mutants correspondingly increased. The increase in total plant biomass in transgenic maize with altered AGP large subunit (Sh2r6hs) indicates that the gene increased AGPase activity and enhanced the sink strength, leading to increased availability and utilization of additional resources (Smidansky et al., 2002). Trehalose feeding activates AGPase *via* post-translational redox modification and the stimulation of starch synthesis in *A. thaliana* leaves (Kolbe et al., 2005). Here, we analyzed the changes in starch and soluble sugar contents in *VaAPL1*-overexpressing *A. thaliana* plants after LT stress. Our results demonstrated that starch, sucrose, glucose, and fructose contents in *VaAPL1*-overexpressing *A. thaliana* plants were higher than those of wild-type plants under LT stress (Figures 3C–F). SuSy is one of the important enzymes that catalyzes the reversible reaction of sucrose and uridine diphosphate (UDP) into uridine diphosphate glucose (UDP-glucose) and fructose to regulate cell osmotic potential response to abiotic stress in plants (Koch, 2004). In present work, the SuSy genes—*AtSUS3*, *AtSUS4*, *SlSUS1*, and *SlSUS4*—were up-regulated (Figures 4F,G, 6G,H). This result is consistent with that of Duan et al. (2021), who reported that *SlSUS1* and *SlSUS4* were up-regulated under polyethylene glycol stress. *AtSUS3* and *AtSUS4* have also been reported to have up-regulated expression under water deprivation, and many studies have demonstrated that freezing stress acts the same as dehydration stress (Baud et al., 2004). Moreover, some genes involved in starch hydrolysis, such as *AtAMY1*, *AtBAM1*, *AtBAM3*, *SlAGB1*, *SlAGB3*, *SlBAM1*, *SlBAM8*, and *SlAMY3*, were up-regulated in *VaAPL1*-overexpressing *A. thaliana* plants and OEs after LT treatment, respectively (Figures 4A–C, 6A–D,F). Among these, *AtAMY1* is strongly induced by biotic and abiotic stresses during starch hydrolysis (Doyle et al., 2010). Hence, starch is converted into soluble sugars to ensure osmotic balance under LT conditions (Krasensky and Jonak, 2012). Our data indicated that overexpression of *VaAPL1* not only increased the starch content in OEs but also increased the sucrose content under LT stress (Figures 5G,H). In addition, the glucose content in the OE leaves also increased (Figure 5I). These substances can alter the osmotic potential of plant cells and improve their LT tolerance. The transcriptome analysis of OE leaves showed that the expression of two peroxisomal butyrate-CoA ligase AAE11 genes and a peroxidase 52 (*Prx52*) gene was positively correlated with antioxidant enzyme activity (Figures 5F, 7B). In addition, *AtPrx52* was found to respond to abiotic stress treatments (Sung et al., 2019). Genes related to sugar metabolism, such as sucrose, raffinose, and galactose synthesis genes were up-regulated (Figure 8). These genes are involved in soluble sugar synthesis to stabilize the cell osmotic potential and maintain positive metabolic processes. These results all further indicate that *VaAPL1* overexpression increases sucrose content, and sucrose, raffinose, and that galactose synthesis genes are positively regulated to enhance tomato plant LT tolerance.

Ca^2+^ has been shown to sense LT stress and interact with target proteins, thereby regulating the expression of stress-responsive genes (Chen et al., 2015). There are three main Ca^2+^ sensor proteins: calcium-dependent protein kinase (CDPK), calmodulin (CaMCML), and calcineurin B-like protein (CBL; Luan et al., 2002). CDPKs are involved in LT signal transduction. OsCPK7/OsCDPK13 is activated by LT stress, and overexpression of either OsCPK7/OsCDPK13 or OsCPK13/OsCDPK7 enhances LT tolerance in rice (Albrecht et al., 2001). CBL-interacting protein kinase 3 (CIPK3) and CIPK7 also participate in the response to LT stress (Huang et al., 2011). CBL proteins are a group of plant calcium sensors that interact exclusively with CIPK proteins (Chen et al., 2015). Forty-seven DEGs encoding CIPK were also identified in the LT-resistant genotypes of Bermuda grass (Chen et al., 2015). Huang et al. (2011) reported that *AtCBL1* was involved in LT stress in *A. thaliana*. Wang and Li (2006) reported that an increase in Ca^2+^ concentration contributes to the maintenance of plasma membrane integrity under stress conditions. In the present study, our transcriptome sequencing results showed that some CDPK and CaMCML genes were up-regulated in the Ca^2+^ signaling pathway in OE leaves after LT stress (Figure 8). This result also indicates that CDPK and CaMCML contribute to LT tolerance in OEs. Auxin signaling is involved in the adaptive response to oxidative stress and salinity in *A. thaliana* (Iglesias et al., 2011). In rice, IAA levels were increased 1.2–1.6-fold following LT treatment at 4°C (Du et al., 2013). Similarly, our transcriptome results revealed that *AUX/IAA* and *GH3* of the auxin pathway were up-regulated in OEs under LT stress (Figure 8). The expression of these genes may contribute to the LT tolerance in OEs. Previous reports have shown that *OsGH3-2* overexpression increases LT tolerance by reducing the free IAA content, alleviating oxidative damage, and decreasing membrane permeability in rice (Aslam et al., 2020). Overall, the expression of these genes directly or indirectly causes cell wall reinforcement, stomatal closure, and defense-related gene expression to enhance LT tolerance in OEs (Figure 8).

## Conclusion

**Figure 9:**
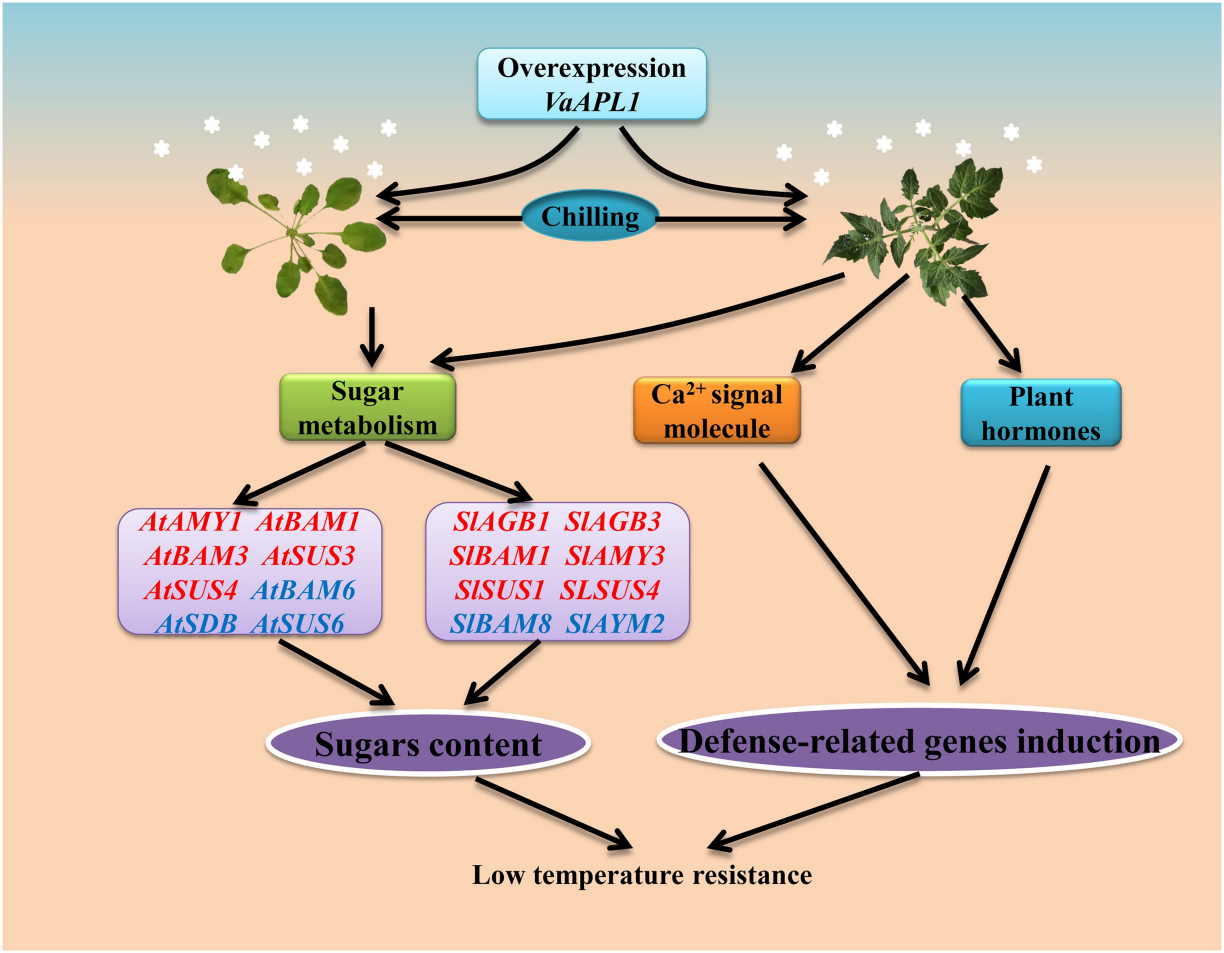
Overexpression of *VaAPL1* improved the LT tolerance of *A. thaliana* and tomato plants. Red and blue colors indicate up- and down-regulated genes, respectively. Briefly, starch degradation and sucrose synthesis-related genes were up-regulated in *VaAPL1*-overexpressing *A. thaliana* plants and OEs after LT stress. The transcriptome data of OEs showed that sucrose and glucose synthesis-related genes were up-regulated during sugar metabolism. Differential expression of these genes increases the contents of sucrose and glucose in OE leaves. Moreover, Ca^2+^ signals and hormone signals in OEs also participate in inducing the expression of defense-related genes. These results ultimately enhance the LT tolerance for *VaAPL1*-overexpressing *A. thaliana* plants and OEs.

In transgenic *A. thaliana* and OEs, *VaAPL1* ensures high levels of soluble sugars to maintain cell osmotic potential and also indirectly promotes ROS scavenging by enhancing the activities of antioxidant enzymes under LT. Moreover, the expression levels of synthesis-related genes involved in the sugar metabolism (such as trehalose, raffinose, and galactose) were up-regulated in OEs, which allows carbohydrates to maintain the stability of cell osmotic potential and enhance LT resistance. Finally, the expression of key genes of the plant hormone signaling pathway and Ca^2+^ signaling pathway were induced and participated in LT resistance (Figure 9). These results provide the basic information required for exploring the roles of *VaAPL1* in LT stress in grapes.

## Data Availability Statement

The datasets presented in this study can be found in online repositories. The names of the repository/repositories and accession number(s) can be found at: https://www.ncbi.nlm.nih.gov/, BioProject PRJNA703431.

## Author Contributions

BC conceived and designed the experiments. GL and JM wrote the manuscript and analyzed data for this work. YL, PW, SJ, and HW contributed to experiments. All authors read and approved the manuscript.

## Funding

This work was financially supported by the Fuxi Foundation of Gansu Agricultural University (no. Gaufx-03J02) and Science and Technology Major Project of Gansu Province (18ZD2NA006).

## Conflict of Interest

The authors declare that the research was conducted in the absence of any commercial or financial relationships that could be construed as a potential conflict of interest.

## Publisher’s Note

All claims expressed in this article are solely those of the authors and do not necessarily represent those of their affiliated organizations, or those of the publisher, the editors and the reviewers. Any product that may be evaluated in this article, or claim that may be made by its manufacturer, is not guaranteed or endorsed by the publisher.

## Footnotes

^1^ https://www.arabidopsis.org/

^2^ https://solgenomics.net/tools/blast/

^3^ http://rice.uga.edu/

^4^ https://www.rosaceae.org/

^5^ http://citrus.hzau.edu.cn/

^6^ https://evolgenius.info//evolview-v2/#mytrees/1/1

## Abbreviations

ROS: Reactive oxygen species
AGPase: ADP-glucose pyrophosphorylase
LT: Low temperature
Col: *Arabidopsis thaliana* ecotype Columbiam
REC: Relative electrolyte leakage
SOD: Superoxide dismutase
POD: Peroxidase
CAT: Catalase
SuSy: Sucrose synthase
AMY: α-amylases
BAM: β-amylase
H_2_O_2_: Hydrogen peroxide
O_2_^−^: Superoxide anion radical
AUX/IAA: Auxin-responsive protein
GH3: Auxin responsive protein
qRT-PCR: Quantitative real-time polymerase chain reaction
WT: Wild-type tomato plants
OEs: *VaAPL1*-overexpressing tomato plants
ML: Maximum likelihood
CDS: Coding sequence
NBT: Nitroblue tetrazolium
DAB: 3, 3′-diaminobenzidine
DEGs: Differentially expressed genes
FDR: False discover rate
KEGG: Kyoto encyclopedia of genes and genomes
SD: Standard deviation
SDB: Starch debranching enzyme
SBE: Starch branching enzyme
CDPK: Calcium-dependent protein kinase
CaMCML: Calmodulin
CBL: Calcineurin B-like protein
CIPK: CBL-interacting protein kinases

## References

[ref1] Abdellatif B. Li J. Miroslav O. (2011). *Arabidopsis thaliana* mutants lacking ADP-glucose pyrophosphorylase accumulate starch and wild-type ADP-glucose content: further evidence for the occurrence of important sources, other than ADP-glucose pyrophosphorylase, of ADP-glucose linked to leaf starch. Plant Cell Physiol. 52, 1162–1176. doi: 10.1093/pcp/pcr067, PMID: 21624897

[ref2] Akihiro T. Mizuno K. Fujimura T. (2005). Gene expression of ADP-glucose pyrophosphorylase and starch contents in rice cultured cells are cooperatively regulated by sucrose and ABA. Plant Cell Physiol. 46, 937–946. doi: 10.1093/pcp/pci101, PMID: 15821022

[ref3] Albrecht V. Ritz O. Linder S. Harter K. Kudla J. (2001). The NAF domain defines a novel protein–protein interaction module conserved in Ca^2+^-regulated kinases. EMBO J. 20, 1051–1063. doi: 10.1093/emboj/20.5.1051, PMID: 11230129 PMC145464

[ref4] Anna L. Teemu P. Tuula J. (2016). Osmolality and non-structural carbohydrate composition in the secondary phloem of trees across a latitudinal gradient in Europe. Front. Plant Sci. 7:726. doi: 10.3389/fpls.2016.00726, PMID: 27313582 PMC4887491

[ref5] Aslam M. Sugita K. Qin Y. Rahman A. (2020). Aux/IAA14 regulates microRNA-mediated cold stress response in *Arabidopsis* roots. Int. J. Mol. Sci. 21:8441. doi: 10.3390/ijms21228441 PMC7697755 33182739

[ref6] Baud S. Vaultier M. Rochat C. (2004). Structure and expression profile of the sucrose synthase multigene family in *Arabidopsis*. J. Exp. Bot. 55, 397–409. doi: 10.1093/jxb/erh047, PMID: 14739263

[ref7] Bennett J. Hondred D. Register J. (2015). Keeping qRT-PCR rigorous and biologically relevant. Plant Cell Rep. 34, 1–3. doi: 10.1007/s00299-014-1692-6, PMID: 25304620

[ref8] Bolouri-Moghaddam M. Roy K. Rolland F. Li X. Rolland F. Ende W. (2010). Sugar signaling and antioxidant network connections in plant cells. FEBS J. 277, 2022–2037. doi: 10.1111/j.1742-4658.2010.07633.x, PMID: 20412056

[ref9] Chen L. Fan J. Hu L. Xie Y. Zhang Y. Lou Y.. (2015). A transcriptomic analysis of bermudagrass (*Cynodon dactylon*) provides novel insights into the basis of low temperature tolerance. BMC Plant Biol. 15:216. doi: 10.1186/s12870-015-0598-y, PMID: 26362029 PMC4566850

[ref10] Chinnusamy V. Zhu J. Zhu J. (2007). Cold stress regulation of gene expression in plants. Trends Plant Sci. 12, 444–451. doi: 10.1016/j.tplants.2007.07.002 17855156

[ref11] Cook F. Fahy B. Trafford K. (2012). A rice mutant lacking a large subunit of ADP-glucose pyrophosphorylase has drastically reduced starch content in the culm but normal plant morphology and yield. Funct. Plant Biol. 39, 1068–1078. doi: 10.1071/FP12186, PMID: 32480856

[ref12] Crevillén P. Ballicora M. Mérida A. Preiss J. Romero J. (2003). The different large subunit isoforms of *Arabidopsis thaliana* ADP-glucose pyrophosphorylase confer distinct kinetic and regulatory properties to the heterotetrameric enzyme. J. Biol. Chem. 278, 28508–28515. doi: 10.1074/jbc.M304280200, PMID: 12748181

[ref13] Czarnocka W. Karpiński S. (2018). Friend or foe? Reactive oxygen species production, scavenging and signaling in plant response to environmental stresses. Free Radical Biol. Med. 122, 4–20. doi: 10.1016/j.freeradbiomed.2018.01.011 29331649

[ref14] Dahiya D. Saini R. Saini H. Devi A. (2017). Sucrose metabolism: controls the sugar sensing and generation of signalling molecules in plants. J. Pharma. Phytochem. 6, 1563–1572.

[ref15] Doyle E. Lane A. Sides J. Mudgett M. Monroe J. (2010). An *α*-amylase (At4g25000) in *Arabidopsis* leaves is secreted and induced by biotic and abiotic stress. Plant Cell Environ. 30, 388–398. doi: 10.1111/j.1365-3040.2006.01624.x, PMID: 17324226

[ref16] Du H. Liu H. Xiong L. (2013). Endogenous auxin and jasmonic acid levels are dierentially modulated by abiotic stresses in rice. Front. Plant Sci. 4:397. doi: 10.3389/fpls.2013.00397, PMID: 24130566 PMC3793129

[ref17] Duan Y. Yang L. Zhu H. Zhou J. Sun H. Gong H. (2021). Structure and expression analysis of sucrose phosphate synthase, sucrose synthase and invertase gene families in *Solanum lycopersicum*. Int. J. Mol. Sci. 22:4698. doi: 10.3390/ijms22094698, PMID: 33946733 PMC8124378

[ref18] Dusenge M. Duarte A. Way D. (2019). Plant carbon metabolism and climate change: elevated CO_2_ and temperature impacts on photosynthesis, photorespiration and respiration. New Phytol. 221, 32–49. doi: 10.1111/nph.15283, PMID: 29983005

[ref19] Fareen S. Yusuf M. Faizan M. Faraz A. Hayat S. (2016). Role of sugars under abiotic stress. Plant Physiol. Biochem. 109, 54–61. doi: 10.1016/j.plaphy.2016.09.005 27639065

[ref20] Feng H. Ma N. Meng X. Zhang S. Wang J. Chai S. (2013). A novel tomato MYC-type ICE1-like transcription factor, SlICE1a, confers cold, osmotic and salt tolerance in transgenic tobacco. Plant Physiol. Biochem. 73, 309–320. doi: 10.1016/j.plaphy.2013.09.014, PMID: 24184451

[ref21] Fritzius T. Aeschbacher R. Wiemken A. Wingler A. (2001). Induction of *ApL3* expression by trehalose complements the starch-deficient Arabidopsis mutant adg2-1 lacking ApL1, the large subunit of ADP-glucose pyrophosphorylase. Plant Physiol. 126, 883–889. doi: 10.1104/pp.126.2.883, PMID: 11402215 PMC111177

[ref22] Gamm M. Héloir M. Bligny R. Vaillant-Gaveau N. Trouvelot S. Alcaraz G.. (2011). Changes in carbohydrate metabolism in *Plasmopara viticola*-infected grapevine leaves. Mol. Plant Microbe Interact 24, 1061–1073. doi: 10.1094/MPMI-02-11-0040, PMID: 21649510

[ref23] Georgelis N. Braun E. Shaw J. Hannah L. (2007). The two agpase subunits evolve at different rates in angiosperms, yet they are equally sensitive to activity-altering amino acid changes when expressed in bacteria. Plant Cell 19, 1458–1472. doi: 10.1105/tpc.106.049676, PMID: 17496118 PMC1913735

[ref24] Hoermiller I. Naegele T. Augustin H. Stutz S. Weckwerth W. Heyer A. (2017). Subcellular reprogramming of metabolism during cold acclimation in *Arabidopsis thaliana*. Plant Cell Environ. 40, 602–610. doi: 10.1111/pce.12836, PMID: 27642699

[ref25] Hou L. Ehrlich M. Thormählen I. Lehmann M. Krahnert I. Obata T.. (2019). NTRC plays a crucial role in starch metabolism, redox balance, and tomato fruit growth. Plant Physiol. 181, 976–992. doi: 10.1104/pp.19.00911, PMID: 31527089 PMC6836810

[ref26] Huang C. Ding S. Zhang H. Du H. An L. (2011). *CIPK7* is involved in cold response by interacting with *CBL1* in *Arabidopsis thaliana*. Plant Sci. 181, 57–64. doi: 10.1016/j.plantsci.2011.03.011 21600398

[ref27] Iglesias M. Terrile M. Casalongué C. (2011). Auxin and salicylic acid signalings counteract the regulation of adaptive responses to stress. Plant Signal Behav. 6, 452–454. doi: 10.4161/psb.6.3.14676, PMID: 21358272 PMC3142437

[ref28] Jia M. Li X. Wang W. Li T. Dai Z. Chen Y.. (2022). SnRK2 subfamily I protein kinases regulate ethylene biosynthesiss by phosphorylating HB transcription factors to induce *ACO1* expression in apple. New Phytol. 234, 1262–1277. doi: 10.1111/nph.18040, PMID: 35182082 PMC9314909

[ref29] Jiang H. Lei T. Wei X. He B. (2015). Changes of sugar contents in different tissues and cell structure in two grape (*Vitis vinifera* L.) varieties under low temperature stress. J. Fruit Sci. 32, 604–611. doi: 10.13925/j.cnki.gsxb.20140464

[ref30] Kathleen W. Helge K. Twan R. (2008). ADP-glucose pyrophosphorylase-deficient pea embryos reveal specific transcriptional and metabolic changes of carbon-nitrogen metabolism and stress responses1. Plant Physiol. 149, 395–411. doi: 10.1104/pp.108.129940, PMID: 18987213 PMC2613696

[ref31] Keunen E. Peshev D. Vangronsveld J. Denende W. Cuypers A. (2013). Plant sugars are crucial players in the oxidative challenge during abiotic stress: extending the traditional concept. Plant Cell Environ. 36, 1242–1255. doi: 10.1111/pce.12061, PMID: 23305614

[ref32] Koch K. (2004). Sucrose metabolism: regulatory mechanisms and pivotal roles in sugar sensing and plant development. Curr. Opin. Plant Biol. 7, 235–246. doi: 10.1016/j.pbi.2004.03.014, PMID: 15134743

[ref33] Kolbe A. Tiessen A. Schluepmann H. Paul M. Ulrich S. Geigenberger P. (2005). Trehalose 6-phosphate regulates starch synthesis via posttranslational redox activation of ADP-glucose pyrophosphorylase. Proc. Natl. Acad. Sci. 102, 11118–11123. doi: 10.1073/pnas.0503410102, PMID: 16046541 PMC1180623

[ref34] Krasensky J. Jonak C. (2012). Drought, salt, and temperature stress-induced metabolic rearrangements and regulatory networks. J. Exp. Bot. 63, 1593–1608. doi: 10.1093/jxb/err460, PMID: 22291134 PMC4359903

[ref35] Lee S. K. Hwang S. K. Han M. (2007). Identification of the ADP-glucose pyrophosphorylase isoforms essential for starch synthesis in the leaf and seed endosperm of rice (*Oryza sativa* L.). Plant Mol. Biol. 65, 531–546. doi: 10.1007/s11103-007-9153-z, PMID: 17406793

[ref36] Liang G. P. Ma Z. H. Lu S. X. Ma W. F. Feng L. D. Mao J.. (2022). Temperature-phase transcriptomics reveals that hormones and sugars in the phloem of grape participate in tolerance during cold acclimation. Plant Cell Rep. 22, 1–17. doi: 10.1007/s00299-022-02862-1, PMID: 35316376

[ref37] Livak K. Schmittgen T. (2001). Analysis of relative gene expression data using real-time quantitative and the 2^-ΔΔCT^ method. Methods 25, 402–408. doi: 10.1006/meth.2001.1262 11846609

[ref38] Lloyd J. Kossmann J. (2015). Transitory and storage starch metabolism: two sides of the same coin? Curr. Opinion Biotechnol. 32, 143–148. doi: 10.1016/j.copbio.2014.11.026, PMID: 25559079

[ref39] Luan S. Kudla J. Rodriguez-Concepcion M. Yalovsky S. Gruissem W. (2002). Calmodulins and calcineurin B-like proteins: calcium sensors for specific signal response coupling in plants. Plant Cell 14, S389–S400. doi: 10.1105/tpc.001115, PMID: 12045290 PMC151268

[ref40] Ma X. Chen C. Yang M. Dong X. Lv W. Meng Q. (2018). Cold-regulated protein (SlCOR413IM1) confers chilling stress tolerance in tomato plants. Plant Physiol. Biochem. 124, 29–39. doi: 10.1016/j.plaphy.2018.01.003, PMID: 29331923

[ref41] Ma X. Gai W. X. Li Y. Yu Y. N. Ali M. Gong Z. H. (2022). The CBL-interacting protein kinase CaCIPK13 positively regulates defence mechanisms against cold stress in pepper. J. Exp. Bot. 73, 1655–1667. doi: 10.1093/jxb/erab505, PMID: 35137060

[ref42] Maruyama K. Urano K. Yoshiwara K. Morishita Y. Sakurai N. Suzuki H.. (2014). Integrated analysis of the effects of cold and dehydration on rice metabolites, phytohormones, and gene transcripts. Plant Physiol. 164, 1759–1771. doi: 10.1104/pp.113.231720, PMID: 24515831 PMC3982739

[ref43] Meng Q. Zhang W. Hu X. Chen L. Dai X. Qu H.. (2020). Two ADP-glucose pyrophosphorylase subunits, *OsAGPL1* and *OsAGPS1*, modulate phosphorus homeostasis in rice. Plant J. 104, 1269–1284. doi: 10.1111/tpj.14998, PMID: 32996185

[ref44] Miao H. Sun P. Liu Q. Liu J. Xu B. Jin Z. (2017). The AGPase family proteins in banana: genome-wide identification, phylogeny, and expression analyses reveal their involvement in the development, ripening, and abiotic/biotic stress responses. Int. J. Mol. Sci. 18:1581. doi: 10.3390/ijms18081581, PMID: 28757545 PMC5577994

[ref45] Morelli R. Russo-Volpe S. Bruno N. Lo S. R. (2003). Fenton-dependent damage to carbohydrates: free radical scavenging activity of some simple sugars. J. Agric. Food Chem. 51, 7418–7425. doi: 10.1021/jf030172q, PMID: 14640593

[ref46] Mugford S. Fernandez O. Brinton J. Flis A. Krohn N. Encke B.. (2014). Regulatory properties of ADP glucose pyrophosphorylase are required for adjustment of leaf starch synthesis in different photoperiods. Plant Physiol. 166, 1733–1747. doi: 10.1104/pp.114.247759, PMID: 25293961 PMC4256850

[ref47] Pérez L. Soto E. Villorbina G. Bassie L. Medina V. Muñoz P.. (2018). CRISPR/Cas9-induced monoallelic mutations in the cytosolic AGPase large subunit gene APL2 induce the ectopic expression of APL2 and the corresponding small subunit gene *APS2b* in rice leaves. Transgenic Res. 27, 423–439. doi: 10.1007/s11248-018-0089-7, PMID: 30099722

[ref48] Rodrigo-Moreno A. Poschenrieder C. Shabala S. (2013). Transition metals: a double edge sward in ROS generation and signaling. Plant Signal Behav. 8:e23245. doi: 10.4161/psb.23425, PMID: 23333964 PMC3676510

[ref49] Rosti S. Fahy B. Denyer K. (2007). A mutant of rice lacking the leaf large subunit of ADP-glucose pyrophosphorylase has drastically reduced leaf starch content but grows normally. Funct. Plant Biol. 34, 480–489. doi: 10.1071/FP06257, PMID: 32689377

[ref50] Salamone P. Greene T. Kavakli I. Okita T. (2000). Isolation and characterization of a higher plant ADP-glucose pyrophosphorylase small subunit homotetramer. FEBS Letter 482, 113–118. doi: 10.1016/s0014-5793(00)01985-2, PMID: 11018533

[ref51] Smidansky E. Clancy M. Meyer F. Lanning S. Blake N. Talbert L.. (2002). Enhanced ADP-glucose pyrophosphorylase activity in wheat endosperm increases seed yield. Proc. Natl. Acad. Sci. 99, 1724–1729. doi: 10.1073/pnas.022635299, PMID: 11830676 PMC122258

[ref52] Smith A. Zeeman S. (2006). Quantification of starch in plant tissues. Nat. Protoc. 1, 1342–1345. doi: 10.1038/nprot.2006.232, PMID: 17406420

[ref53] Sun X. Zhang L. Wong D. Wang Y. Zhu Z. Xu G.. (2019). The ethylene response factor *VaERF092* from Amur grape regulates the transcription factor *VaWRKY33*, improving cold tolerance. Plant J. 99, 988–1002. doi: 10.1111/tpj.14378, PMID: 31063661

[ref54] Sung Y. Lee I. Shim D. Lee K. Kim Y. (2019). Transcriptomic changes in sweetpotato peroxidases in response to infection with the root-knot nematode *Meloidogyne incognita*. Mol. Biol. Rep. 46, 4555–4564. doi: 10.1007/s11033-019-04911-7, PMID: 31222458

[ref55] Takashi O. Francisco P. Takayuki S. Tatsuro H. Tomio T. Hikaru S.. (2005). Expression profiling of genes involved in starch synthesis in sink and source organs of rice. J. Exp. Bot. 56, 3229–3244. doi: 10.1093/jxb/eri292, PMID: 16275672

[ref56] Thitisaksakul M. Jimenez R. Arias M. Beckles D. (2012). Effects of environmental factors on cereal starch biosynthesis and composition. J. Cereal Sci. 56, 67–80. doi: 10.1016/j.jcs.2012.04.002

[ref57] Ulfat A. Mehmood A. Ahmad K. Ul-Allah S. (2021). Elevated carbon dioxide offers promise for wheat adaptation to heat stress by adjusting carbohydrate metabolism. Physiol. Mol. Biol. Plants 27, 2345–2355. doi: 10.1007/s12298-021-01080-5 34744370 PMC8526630

[ref58] Wang L. Li S. (2006). Salicylic acid-induced heat or cold tolerance in relation to Ca2^+^ homeostasis and antioxidant systems in young grape plants. Plant Sci. 170, 685–694. doi: 10.1016/j.plantsci.2005.09.005

[ref59] Wiberley-Bradford A. Busse J. Bethke P. (2016). Temperature-dependent regulation of sugar metabolism in wild-type and low-invertase transgenic chipping potatoes during and after cooling for low-temperature storage. Postharvest Biol. Technol. 115, 60–71. doi: 10.1016/j.postharvbio.2015.12.020

[ref60] Wolfe J. Bryant G. (1999). Freezing, drying, and/or vitrification of membrane- solute-water systems. Cryobiology 39, 103–129. doi: 10.1006/cryo.1999.2195 10529304

[ref61] Xu W. Li R. Zhang N. Ma F. Jiao Y. Wang Z. (2014). Transcriptome profiling of *Vitis amurensis*, an extremely cold-tolerant Chinese wild *Vitis species*, reveals candidate genes and events that potentially connected to cold stress. Plant Mol. Biol. 86, 527–541. doi: 10.1007/s11103-014-0245-2, PMID: 25190283

[ref62] Yan L. Zhai Q. Wei J. Li S. Wang B. Huang T.. (2013). Role of tomato lipoxygenase D in wound-induced jasmonate biosynthesis and plant immunity to insect herbivores. PLoS Genet. 9:e1003964. doi: 10.1371/journal.pgen.1003964, PMID: 24348260 PMC3861047

[ref63] Zhang L. Zhao T. Sun X. Wang Y. Du C. Zhu Z.. (2019). Overexpression of *VaWRKY12*, a transcription factor from *Vitis amurensis* with increased nuclear localization under low temperature, enhances cold tolerance of plants. Plant Mol. Biol. 100, 95–110. doi: 10.1007/s11103-019-00846-6, PMID: 31011887

[ref64] Zhao Y. Luo L. Xu J. Xin P. Guo H. Wu J. (2018). Malate transported from chloroplast to mitochondrion triggers production of ROS and PCD in *Arabidopsis thaliana*. Cell Res. 28, 448–461. doi: 10.1038/s41422-018-0024-8, PMID: 29540758 PMC5939044

[ref65] Zhao L. Yang T. Xing C. Dong H. Qi K. Gao J.. (2019). The β-amylase *PbrBAM3* from pear (*Pyrus betulaefolia*) regulates soluble sugar accumulation and ROS homeostasis in response to cold stress. Plant Sci. 287:110184. doi: 10.1016/j.plantsci.2019.110184, PMID: 31481191

[ref66] Zhou Y. Chen Y. Tao X. Cheng X. Wang H. (2016). Isolation and characterization of cDNAs and genomic DNAs encoding ADP-glucose pyrophosphorylase large and small subunits from sweet potato. Mol. Gen. Genomics. 291, 609–620. doi: 10.1007/s00438-015-1134-3, PMID: 26499957

[ref67] Zhu J. (2016). Abiotic stress signaling and responses in plants. Cell 167, 313–324. doi: 10.1016/j.cell.2016.08.029, PMID: 27716505 PMC5104190

