# Supplementary material for: VaAPL1 Promotes Starch Synthesis to Constantly Contribute to Soluble Sugar Accumulation, Improving Low Temperature Tolerance in Arabidopsis and Tomato
Source: Front Plant Sci. 2022 Jun 22;13:920424. doi: 10.3389/fpls.2022.920424 (PMC9257282; doi:10.3389/fpls.2022.920424)
Supplement: Supplementary file 1 [file Data_Sheet_1.PDF]

Supplementary Figures and Tables

1. Supplementary Figures

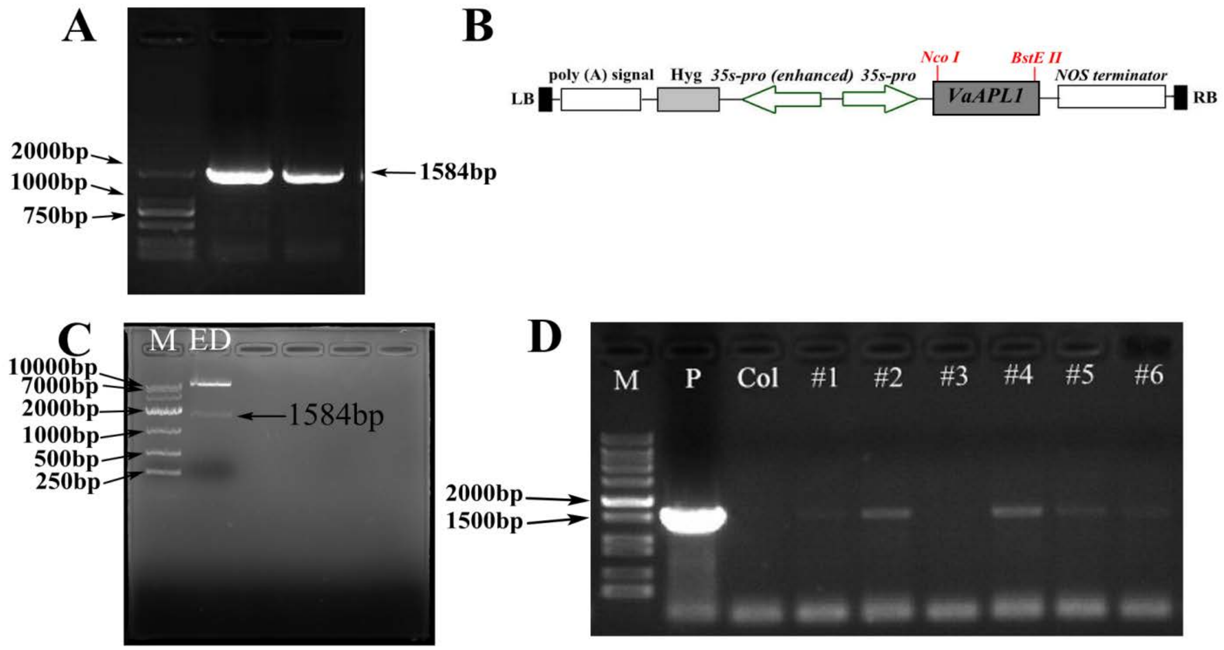

Supplementary Figure S1 The *VaAPL1* gene cloned, recombinant plasmid pCAMBIA1301-*VaAPL1* constructed, and the *VaAPL1*-overexpressing *A. thaliana* plants identified. A The coding sequences of *VaAPL1* was cloned. **B** The *VaAPL1* was inserted into position of pCAMBIA1301 plasmid. **C** Recombinant plasmid pCAMBIA1301-*VvBAM1* was confirmed by double enzyme digestion. ED indicates recombinant plasmid was digested by *Nco* I and *BstE* II restriction enzymes. M indicates DNA maker ladder (DL 10k bp). **D** PCR identification of *VaAPL1*-overexpressing *A. thaliana* plants. M for marker, P for positive control, Col for wild-type, and #1 – #6 were *VaAPL1*-overexpressing T_1 *A. thaliana* positive plants.

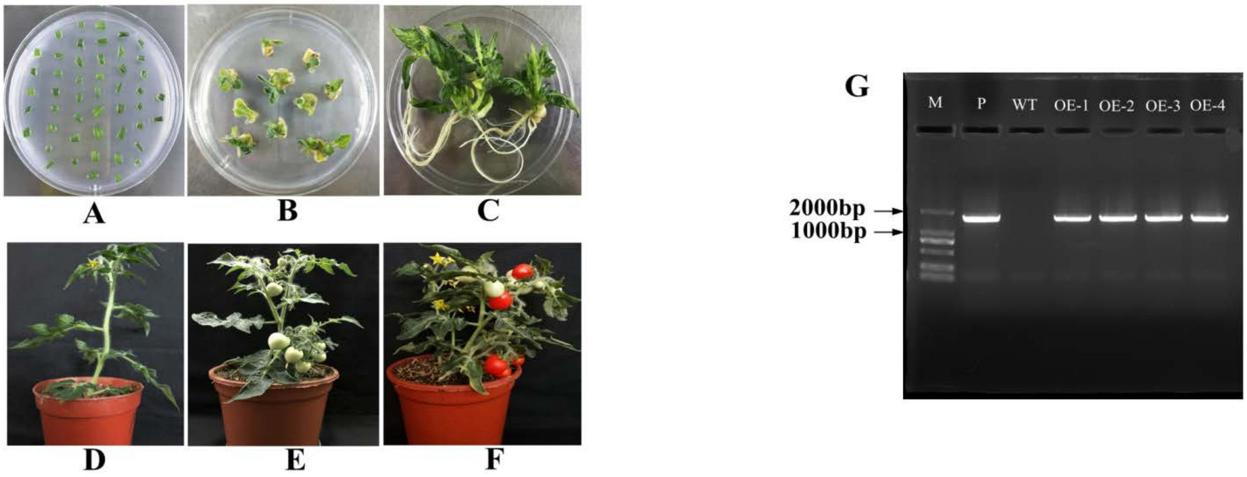

Supplementary Figure S2 The regeneration of tomato and identification of OEs. A Tomato cotyledon pre-cultures stage**. B** Explant buds formation stage on hygromycin resistant medium. **C** Formation roots of OEs on hygromycin resistant medium. **D** The OEs vegetative growth period. **E** The OEs young fruit period. **F** The OEs fruit maturity period**. G** PCR identified OEs. M for marker, P for positive control, WT for wild-type, and OE-1 – OE-4 were four *VaAPL1*-overexpressing T_0 generation positive tomato plants.

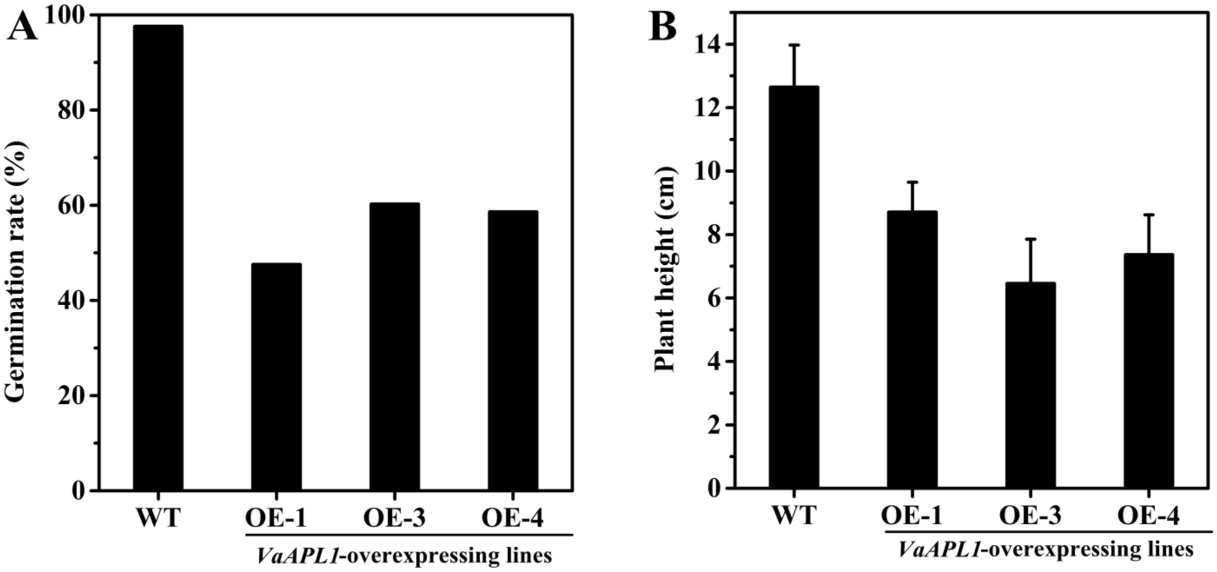

Supplementary Figure S3 The seeds germination rate of T_3 generation OEs and plant height. A Seeds germination rate of WT and OEs at normal temperature. **B** Plant high of 6-week-old WT and OEs at 25 °C.

2. Supplementary Tables

Supplementary Table S1 The ID of *APL* genes of different species

Plants species	Gene Name	Gene ID	GenBank Accession
Arabidopsis thaliana	*AtAPL1*	AT5G19220. 1	NM_121927
	AtAPL2	AT1G27680. 1	NM_102533
	AtAPL3	AT4G39210. 1	NM_120081
	AtAPL4	AT2G21590. 1	NM_127730
Vitis amurensis	*VaAPL1*	GSVIVT01017911001	
Malus domestica	*MdAPL1*	MDP0000165351	NM_001328845.1
	MdAPL2	MDP0000227577	XM_008387677.3
	MdAPL3	MDP0000231298	XM_008342710.3
	MdAPL4	MDP0000252369	XM_029106623.1
	MdAPL5	MDP0000256619	XM_008389820.3
	MdAPL6	MDP0000258518	XM_029102026.1
	MdAPL7	MDP0000293184	XM_029091606.1
	MdAPL8	MDP0000294583	XM_008379059.3
	MdAPL9	MDP0000722046	XM_008379058.3
	MdAPL10	MDP0000884993	XM_008344054.3

	CitAPL1	Orange1.1g013483m	XM_006473471.3
Citrus sinensis	*CitAPL2*	Orange1.1g015713m	XM_006491376.3
	CitAPL3	Orange1.1g009720m	XM_006489265.3
	PpAPL1	Prupe.1G153800.1	XM_007222459.2
Prunus persica	*PpAPL2*	Prupe.1G376200.1	XM_007201099.2
	PpAPL3	Prupe.8G093900.1	XM_007222609.2
	SlAPL1	Solyc01g079790.2.1	NM_001246989.2
Solanum lycopersicum	*SlAPL2*	Solyc01g109790.2.1	NM_001247018.2
	SlAPL3	Solyc07g019440.2.1	NM_001247048.3
	OsAPL1	Os01g44220.1	XM_015776532.2
	OsAPL2	Os03g52460.1	XM_015794884.2
	OsAPL3	Os05g50380.1	XM_015784987.1
Oryza sativa	*OsAPL4*	Os07g13980.1	XM_015789740.2
	OsAPL5	Os08g25734.1	D50317.1
	OsAPL6	Os09g12660.1	EU267957.1

Supplementary Table S2 qRT-PCR primers for expression on analysis of starch and sugar metabolism related genes in *VaAPL1*-overexpressing *A. thaliana* and tomato plants

Gene name	Gene accession NO.	qRT-PCR Primer 5′→3′		Product size (bp)
AtAMY3	AT1G69830	F: CTGGGACGATAGGGCAGTAG	R: GCCTCCATCCATCATACCCA	179
AtAMY1	AT4G25000	F: TCGACACCGGAGGAGATTTT	R: ATCGTCCCATTTCTCACCCA	215
AtBAM6	AT2G32290	F: CAACAGAACCAAAGGCGACA	R: CAACGATTCCCCACCATACG	210
AtBAM1	AT5G47010	F: TGCGAGCAAAACACAAGGAA	R: TCGTCAATCAAGGGACACCA	223
AtSDB	AT5G04360	F: ACTCCGGTGACTGGTTCAAT	R: TTGTGGAGGGTGGCAACTAT	169
AtSUS6	AT1G73370	F: AGGTTGGGGAGATACAGCAG	R: TGCTGACCAAAATAGCCGTG	159
AtSUS4	AT3G43190	F: GCACCTGACCCTTGTACTCT	R: TTGTTTGATGCGTTGGAGCA	192
AtSUS3	AT4G02280	F: CGCTACATCGCGGATACAAG	R: ATCGATGTGGAAACCCGAGA	165
AtActin	AT3G46520	F: ACGGGAGTGATGGTTGGAAT	R: TCACGGTTAGCCTTCGGATT	230
SlAGB1	XM_010327630	F: GGGCTTTTCCCAGATGCAAT	R: ACTCTCCAATCCTCATCCCG	164
SlAGB3	XM_004238722	F: CGTTATGAGGTGGCAGCAAA	R: TACCACATCATACGACGGCA	165
SlAMY3	XM_004238722	F: CAATGAGGGGTCTAGTGCGA	R: CACCTTTGCAGATTCCCCAG	194
SlAMY2	XM_004238354	F: CACTGGTGGAAGGGGTAACA	R: ATTGGCTTAGCTCCCTCGAT	207
SlBAM8	XM_004244394	F: ATCCAAACCCGACTCACCAT	R: TTCCCGTACTGACGAAGTCC	214
SlBAM1	NM_001247627	F: ATGGATGTGTGGTGGGGATT	R: AAGTGTATCGCAACCAAGCG	170
SlSUS	XM_019211274	F: CACGAGCTTTTGGCTGAGTT	R: TTCACACGGACGTATTCCCA	173
SlSUS4	XM_004247925	F: AGGCCAGGTGTTTGGGAATA	R: AATGGGTCGAGGGATTCGTT	266
SlActin	NM_001330119	F: GGCAGACGGAGAGGATATTCA	R: TGACCCATACCCACCATCAC	150

Supplementary Table S3 Summary of reads based on RNA sequencing data obtained from each sample of *VaAPL1*-overexpressing tomato after 24 h LT stress

Samples	Total Reads	Mapped Reads	Unique Mapped Reads	Multiple Map Reads
WT1	48,261,944	41,658,862 (86.32%)	36,515,894 (75.66%)	5,142,968 (10.66%)
WT2	43,050,182	36,990,831 (85.92%)	33,133,379 (76.96%)	3,857,452 (8.96%)
WT3	42,091,928	34,889,156 (82.89%)	31,610,729 (75.10%)	3,278,427 (7.79%)
OE1	44,116,656	27,742,295 (62.88%)	24,975,116 (56.61%)	2,767,179 (6.27%)
OE3	43,924,330	37,670,679 (85.76%)	34,291,472 (78.07%)	3,379,207 (7.69%)
OE4	43,251,944	32,348,998 (74.79%)	29,316,372 (67.78%)	3,032,626 (7.01%)

% ≥ Q30: The percentage of clean reads whose quality score was more than 30.

Supplementary Table S4 The DEGs and annotation information of heatmap

Gene ID	FPKM		*p*-Value	log_2FC	Annotation information
	WT	Transgenic lines			
Solyc01g090697.1	0	2.90858867	1.62E-17	3.313812	3-hydroxyisobutyryl-CoA hydrolase-like protein 1, mitochondrial
Solyc02g081345.1	3.431779	23.0260017	5.90E-12	2.146095	Butyrate--CoA ligase AAE11, peroxisomal
Solyc02g081370.2	1.185862	7.32097233	2.81E-10	2.018064	Butyrate--CoA ligase AAE11, peroxisomal
Solyc02g087180.4	0	1.750159	1.62E-27	4.087305	Mediator of RNA polymerase II transcription subunit 4
Solyc03g007790.3	1.045801	6.07499067	7.52E-21	2.108832	G-type lectin S-receptor-like serine/threonine-protein kinase At5g24080
Solyc03g078570.3	0.87523	16.075544	1.02E-07	2.088729	Ras-related protein RABH1e
Solyc04g009850.4	6.649582	41.8147467	1.87E-13	2.155258	1-aminocyclopropane-1-carboxylate oxidase homolog 1
Solyc04g082155.1	0.176161	17.269847	7.69E-31	4.147649	--
Solyc05g010780.1	0.041247	0.93270133	2.78E-10	2.429288	Putative pentatricopeptide repeat-containing protein At1g13630
Solyc05g012300.1	0.084374	5.10966833	1.03E-14	2.96269	E3 ubiquitin-protein ligase COP1
Solyc05g052280.3	3.853253	26.0582733	1.28E-11	2.139091	Peroxidase 52
Solyc06g048740.3	0.353413	3.42076	6.03E-11	2.295702	Probable LRR receptor-like serine/threonine-protein kinase At3g47570
Solyc06g049030.4	0	2.08915467	5.83E-08	2.122536	RING-H2 finger protein ATL72

Solyc06g076540.1	0	5.81387367	8.39E-13	2.815789	17.7 kDa class I heat shock protein
Solyc06g083650.3	0.040768	5.09675933	2.68E-07	2.01208	GDSL esterase/lipase At5g33370
Solyc07g045000.4	0.795427	8.87062567	9.71E-17	2.515303	Putative Myb family transcription factor At1g14600
Solyc07g063850.3	0.931846	6.08962367	2.30E-13	2.175182	Indole-3-acetic acid-amido synthetase GH3.6
Solyc08g082460.4	0	2.64604733	1.60E-20	3.580025	Probable inactive receptor kinase RLK902
Solyc09g005840.2	1.2746	7.574192	7.24E-16	2.162805	Exocyst complex component EXO70H1
Solyc09g014510.3	1.146365	36.3828617	1.92E-17	3.261837	Chloroplast processing peptidase
Solyc09g098080.4	0.716482	6.54372733	3.27E-09	2.135592	UDP-glycosyltransferase 71E1
Solyc10g011925.1	0	0.97397533	2.64E-08	2.182998	Phenylalanine ammonia-lyase
Solyc10g081205.1	0.002394	0.81803933	7.28E-13	2.81807	--
Solyc11g072470.3	5.26664	36.979423	5.49E-12	2.187033	LOB domain-containing protein 1
Solanum_lycopersicum_newGene_5313	0	2.04229533	5.49E-11	2.583992	Receptor-like protein Cf-9 homolog
Solyc01g008390.3	6.805466	0.84888833	1.60E-09	-2.15025	Ubiquinol oxidase, mitochondrial
Solyc01g105220.3	1.267835	0	1.43E-15	-3.12282	Clathrin interactor EPSIN 3
Solyc01g110510.3	1.09133	0	9.25E-08	-2.08872	--
Solyc02g038816.1	21.47403	1.165266	1.70E-14	-2.93633	Chlorophyll a-b binding protein 7, chloroplastic
Solyc04g082930.3	11.55262	0.201791	5.37E-08	-2.14287	Phosphate transporter PHO1 homolog 1

Solyc05g010060.4	19.67728	3.10171567	1.42E-15	-2.25069	Cytokinin riboside 5'-monophosphate phosphoribohydrolase LOG1
Solyc06g075090.4	33.27799	7.367445	2.95E-17	-2.03152	Pathogenesis-related protein PR-1
Solyc07g006700.1	36.73497	5.30243133	4.27E-09	-2.06406	--
Solyc07g008075.1	3.677555	0.00455133	5.22E-11	-2.58684	Protein trichome birefringence-like 14
Solyc07g008820.3	8.514772	0.03529067	3.45E-89	-5.84476	Probable amino acid permease 7
Solyc07g066020.2	2.198337	0.09786	1.74E-09	-2.27507	Kirola
Solyc08g023660.3	20.72761	1.07622	9.59E-10	-2.37398	Transcription factor TCP2
Solyc08g048390.3	8.197718	0.43179333	4.54E-23	-3.24545	UDP-glucuronate 4-epimerase 2
Solyc08g079440.1	4.361729	0.191121	1.17E-11	-2.59737	--
Solyc08g082120.2	294.4602	43.6407623	3.13E-17	-2.186	Protein LIFEGUARD 4
Solyc09g065610.2	30.86642	0.22054333	6.84E-25	-3.78904	--
Solyc09g074675.1	1.751251	0.01770833	3.04E-10	-2.47835	Transcription factor FAMA
Solyc09g091760.3	3.357226	0.070095	2.39E-09	-2.33761	Retrovirus-related Pol polyprotein from transposon RE1
Solyc10g084125.1	5.610619	0.295119	9.38E-08	-2.09722	Abscisic acid receptor PYL2
Solyc12g095970.3	7.565534	0.92774433	3.65E-08	-2.03265	--
Solanum_lycopersicum_newGene_3964	4.18375	0	5.12E-10	-2.44792	--
